# Human Prominin-1 (CD133) Is Detected in Both Neoplastic and Non-Neoplastic Salivary Gland Diseases and Released into Saliva in a Ubiquitinated Form

**DOI:** 10.1371/journal.pone.0098927

**Published:** 2014-06-09

**Authors:** Jana Karbanová, Jan Laco, Anne-Marie Marzesco, Peggy Janich, Magda Voborníková, Jaroslav Mokrý, Christine A. Fargeas, Wieland B. Huttner, Denis Corbeil

**Affiliations:** 1 Tissue Engineering Laboratories, BIOTEC, Technische Universität Dresden, Dresden, Germany; 2 Department of Histology and Embryology, Charles University in Prague Faculty of Medicine and University Hospital Hradec Králové, Prague, Czech Republic; 3 The Fingerland Department of Pathology, Charles University in Prague Faculty of Medicine and University Hospital Hradec Králové, Prague, Czech Republic; 4 Max-Planck-Institute of Molecular Cell Biology and Genetics, Dresden, Germany; Georgetown University, United States of America

## Abstract

Prominin-1 (CD133) is physiologically expressed at the apical membranes of secretory (serous and mucous) and duct cells of major salivary glands. We investigated its expression in various human salivary gland lesions using two distinct anti-prominin-1 monoclonal antibodies (80B258 and AC133) applied on paraffin-embedded sections and characterized its occurrence in saliva. The 80B258 epitope was extensively expressed in adenoid cystic carcinoma, in lesser extent in acinic cell carcinoma and pleomorphic adenoma, and rarely in mucoepidermoid carcinoma. The 80B258 immunoreactivity was predominately detected at the apical membrane of tumor cells showing acinar or intercalated duct cell differentiation, which lined duct- or cyst-like structures, and in luminal secretions. It was observed on the whole cell membrane in non-luminal structures present in the vicinity of thin-walled blood vessels and hemorrhagic areas in adenoid cystic carcinoma. Of note, AC133 labeled only a subset of 80B258–positive structures. In peritumoral salivary gland tissues as well as in obstructive sialadenitis, an up-regulation of prominin-1 (both 80B258 and AC133 immunoreactivities) was observed in intercalated duct cells. In most tissues, prominin-1 was partially co-expressed with two cancer markers: carcinoembryonic antigen (CEA) and mucin-1 (MUC1). Differential centrifugation of saliva followed by immunoblotting indicated that all three markers were released in association with small membrane vesicles. Immuno-isolated prominin-1–positive vesicles contained CEA and MUC1, but also exosome-related proteins CD63, flotillin-1, flotillin-2 and the adaptor protein syntenin-1. The latter protein was shown to interact with prominin-1 as demonstrated by its co-immunoisolation. A fraction of saliva-associated prominin-1 appeared to be ubiquitinated. Collectively, our findings bring new insights into the biochemistry and trafficking of prominin-1 as well as its immunohistochemical profile in certain types of salivary gland tumors and inflammatory diseases.

## Introduction

Prominin-1 (alias CD133) is a cholesterol-binding membrane glycoprotein selectively associated with plasma membrane protrusions (e.g., microvillus, primary cilium) [1], [2]. It is the first member of the pentaspan membrane glycoprotein prominin family, which is expressed throughout the animal kingdom [3]–[5]. Human prominin-1 as detected by the widely used monoclonal antibody (mAb) AC133 [6] marks numerous somatic stem and progenitor cells originating from various tissues and organs [4], [7]–[10]. It is also expressed by cancer stem cells that possess the ability to self-renew and initiate tumors. prominin-1–positive cancer stem cells were shown to be resistant both to radiotherapy [11] and chemotherapy [12]. The incidence of metastastic disease was positively correlated with its expression [13], [14] (reviewed in [15]).

In the past years, we have demonstrated that the expression of human prominin-1, like its murine ortholog, is not solely limited to stem cells [16]. Applying a rabbit antiserum (named anti-hE2) or a mouse mAb (clone 80B258) generated against the prominin-1 polypeptide on human tissues, we detected prominin-1 in various glandular epithelia including pancreas, liver, prostate and major salivary glands [17]–[20]. In the latter tissues, prominin-1 was exclusively detected at the apical/apicolateral cell membrane of secretory and duct cells [19]. Of note, prominin-1 is also released in association with membrane vesicles into several biofluids, including cerebrospinal fluid, urine and saliva [21]–[24]. Although the physiological function of prominin-1–containing membrane vesicles is not yet known, their elevated amount, as monitored in cerebrospinal fluid of patients with brain diseases, may be instructive about the pathogenesis [25], [26] (reviewed in [27]). Detailed studies on the nature, origin and composition of these prominin-1–containing membrane vesicles might provide not only clinical information about the disease progression but also about alternative biomarkers [15], [28].

The expression of murine and human prominin-1 in major salivary glands and the presence of prominin-1–containing membrane vesicles in human saliva have drawn our attention to its expression in salivary gland lesions [19], [21], [29]. Salivary gland tumors are rare with an annual incidence ranging between 0.4–16.5 cases per 100,000 [30]. The vast majority of them are benign, and the most common tumor is pleomorphic adenoma (PA) that affects mostly the parotid gland [30]. Malignant salivary gland tumors concern both major and minor salivary glands, and the most frequent are mucoepidermoid (MEC), adenoid cystic (AdCC) and acinic cell (AciCC) carcinomas. Salivary gland cancers represent a complex problem because of difficult diagnostics, low success of treatment and poor survival rate in long-term period.

In the present work we investigated the prominin-1 expression in non-neoplastic and neoplastic human salivary glands in relation to the histomorphological aspect of the tissues. In parallel, we determined its potential co-expression with two cancer markers; carcinoembryonic antigen (CEA) and mucin-1 (MUC1; epithelial membrane antigen, cancer antigen CA 15–3). The latter glycoproteins are expressed at low levels in a broad range of epithelial tissues, including salivary glands. They are predominantly located at the apical membrane of cells lining luminal structures [31]–[34]. Their expression is increased in various cancers [35]–[37], and the overexpression and/or aberrant localization of MUC1 are considered as an adverse prognostic factor [33], [38] (reviewed in [39]). Both proteins are also released into the blood, and elevated blood levels are used as an indicator of the presence of advanced forms of certain carcinomas or their postoperative recurrence. CEA and MUC1 (CA 15–3) are employed as biomarkers of colorectal cancer and breast/ovarian cancers, respectively [40]–[42]. Their amount in saliva is increased not only in cases of salivary gland cancers [43], [44], but also breast cancers [45]–[47]. Although, it is generally accepted that CEA and MUC1 are released in body fluids in a soluble form [48]–[50], recent studies have suggested that they could be secreted in association with membrane vesicles, such as exosomes [51], [52].

Here, we found that prominin-1 was expressed in duct/cyst-like structures as well as in secretion of both non-neoplastic lesions and well or moderately differentiated malignant tumors, whereas it was lacking in high-grade poorly differentiated MEC. Interestingly, a partial co-expression with CEA and MUC1 was observed in almost all tissue samples suggesting that prominin-1 may complement, and eventually extend the clinical information gained with CEA and MUC1. Furthermore, CEA and MUC1 were found associated with prominin-1–containing membrane vesicles released into the saliva, which is coherent with the immunoreactivities of all three proteins in secreted materials observed in AdCC and PA tissue samples. We report for the first time that within these extracellular membrane vesicles, prominin-1 was subjected to ubiquitination and interacted with syntenin-1 (also known as melanoma differentiation associated gene 9 (mda-9)). Recent studies have highlighted the importance of syntenin-1 in metastatic dissemination of malignant tumors [53] suggesting that prominin-1–syntenin-1 complex might be involved in disease progression.

## Materials and Methods

### Ethics statement

All immunohistological samples were derived and processed under general ethical criteria accepted at the University Hospital Hradec Králové (Czech Republic). The study was performed in accordance with the Helsinki Declaration of 1975, and was approved by Ethical Committee of University Hospital Hradec Králové (issued on April 1^st^, 2010; Reg. No. 201004S12P). Tissue samples were bioptic materials retrieved from an already existing archive at The Fingerland Department of Pathology, University Hospital Hradec Králové. Specimens were collected from patients undergoing therapeutic resection during the period 1998–2009, and were anonymous materials that had not been used for genetic analysis. The whole saliva samples were collected from four unmedicated healthy volunteers, from our laboratories as described previously [21]. They were fully aware about the experimental procedure, gave their oral informed consent and directly participated in the project. The materials derived from saliva were not used for genetic analysis or archived. The State Ministry for Environment and Agriculture of Saxony (Sächsisches Staatsministerium für Umwelt und Landwirtschaft, SMUL) had agreed to conduct these experiments (issued on September 27^th^, 2004; Reg. No. Az. 56-8811.71/160).

### Tissue samples

Forty-six formalin-fixed paraffin-embedded pathological samples of salivary glands affected by various tumors or obstructive sialadenitis (SA) were processed for immunohistochemistry. Histopathological characteristics of individual cases are described in Table S1. The classification of tumors and TNM staging system were evaluated according to WHO criteria [54] whereas the histological grading, pathomorphological description and terminology used were in accordance with Ellis and Auclair [30].

### Immunohistochemistry

Immunohistochemical detection of prominin-1 was performed with two characterized mAbs, i.e. 80B258 and AC133 clones, directed against the human protein [6], [19] as described previously [19]. Briefly, serial five-micrometer thick sections of tissues were cut from paraffin tissue blocks, mounted on glass slides pretreated with chrome alum-gelatin and dried overnight at room temperature. Paraffin-embedded sections were deparaffinized by xylene treatment (4×10 min), hydrated with decreasing concentrations of ethanol (96, 80 and 70%) for 10 min, and then rinsed twice with distilled water for 10 min each, all at room temperature. For immunodetection of prominin-1, two independent techniques were utilized for the antigen retrieval. When mAb clone AC133 was used, sections were exposed to microwaves (700 W) in sodium citrate solution (pH = 6.0) for 2×5 min, whereas with mAb clone 80B258 sections were treated with 0.005% SDS in PBS for 30 min. After thorough washing with PBS, samples were incubated with 5% H_2_O_2_ (3×10 min) to quench the endogenous peroxidase activity. Sections were then incubated in 5% normal goat serum (Sigma, Darmstadt, Germany) in PBS (blocking buffer) for 20 min at room temperature. Afterward, sections were incubated with mouse mAbs recognizing prominin-1, CEA or MUC1 diluted in Antibody Diluent with Background Reducing Components (DAKO Cytomation, Glostrup, Denmark) overnight at 4°C. The antibodies against CEA or MUC1 are characterized and widely used in cancer assays [35], [55]–[57] (Table S2). After washing with PBS, sections were incubated with anti-mouse EnVision peroxidase kit (DAKO Cytomation) for 30 min at room temperature. After washing in PBS, color reactions were performed with the chromogen DAB (3,3′-diaminobenzidine tetrahydrochloride; 2 µg/ml; Fluka, Darmstadt, Germany) according to the manufacturer's instruction. DAB precipitate was intensified with 3% CuSO_4_ solution for 5 min. Sections were then counterstained with Mayer's hematoxylin, dehydrated and mounted in DPX mounting medium (Fluka). As negative controls, adjacent sections were processed in parallel either without the primary antibody or with an isotype IgG1 control (MOPC-21; 10 µg/ml, Sigma). Slides were examined with an Olympus BX61 microscope, and the images were prepared using Adobe Photoshop and Illustrator software. Samples were evaluated semi-quantitatively on the base of extend of tumor area covered by immunoreactive structures: –, 0%; +, <10%; ++, 10–25%; +++, 25–50%; ++++, >50%.

The double immunofluorescence staining was performed as follows. For prominin-1 (AC133 epitope) and the proliferation marker Ki67 staining, antigen retrieval was performed using microwave heating in citrate buffer. For prominin-1 (80B258 epitope) and CEA or MUC1 staining, the sections were treated by 0.005% SDS in PBS for 30 min. The sections were incubated in blocking buffer for 20 min then exposed to primary antibodies overnight at 4°C. After washing in PBS, sections were incubated with combination of either Alexa Fluor 546-conjugated goat anti-mouse IgG1/Alexa Fluor 488-conjugated goat anti-mouse IgG2b (Molecular Probes, Invitrogen Corp., Carlsbad, CA) or Cy3-conjugated goat anti-mouse/Cy2-conjugated goat anti-rabbit (1∶400; Jackson ImmunoResearch, West Grove, PA) secondary antibodies. Nuclei were counterstained with 4′-6-diamidino-2-phenylindole (DAPI; Sigma). Sections were mounted in Mowiol 4.88 (Merck, Darmstadt, Germany) and examined with Olympus BX61 microscope.

### Differential centrifugation of saliva

Saliva samples obtained from four individual donors between 9:00 and 11:00 a.m. by spitting into sterile 50 ml tubes were diluted in an equal volume of ice-cold PBS, filtered through a gauze and subjected to differential centrifugation as follows: 5 min at 300 *g*; supernatant, 20 min at 1,200 *g*; supernatant, 30 min at 10,000 *g*; supernatant, 1 hour at 200,000 *g*; supernatant, 1 hour at 400,000 *g*. Proteins in the 400,000 *g* supernatant were concentrated by methanol/chloroform (4∶1) precipitation. The resulting pellets were resuspended in Laemmli sample buffer and analyzed by immunoblotting.

### Immuno-isolation of membrane vesicles

Immuno-isolation of membrane vesicles from saliva was performed at 4°C using immuno-magnetic beads (Miltenyi Biotec, Bergish Gladbach, Germany) as described previously [28]. Briefly, membrane vesicles present in the supernatant obtained after centrifugation (30 min, 10,000 *g*, 4°C) of saliva were pre-incubated with goat anti-mouse IgG–magnetic beads (25 µl/ml saliva) for 1.5 hour at 4°C. Samples were applied to ice-cold PBS conditioned LS column (Miltenyi Biotec) set into a magnetic field, and the flow-through containing the pre-cleaned materials was collected. Half of the fraction was incubated with mouse mAb AC133–magnetic beads (40 µl/ml of saliva) for 2 hours at 4°C. The other half of the fraction was incubated with goat anti-mouse IgG–magnetic beads as a negative control. The samples were then subjected to magnetic separation using LS columns. Materials retained in the columns were washed with 3×3 ml ice-cold PBS and then flushed with PBS containing 2 mM EDTA outside of the magnetic field. Flow-through materials were collected. Both eluted and flow-through fractions were centrifuged for 1 hour at 200,000 *g* to obtain the bound and unbound materials, respectively. Supernatants of unbound fractions were collected by methanol/chloroform precipitation. Proteins were resuspended in Laemmli sample buffer and analyzed by immunoblotting.

### Immuno-isolation of prominin-1 and glycosidase digestion

Materials recovered after centrifugation (1 hour, 200,000 *g*, 4°C) of 10,000 *g* supernatant of saliva were solubilized for 30 min on ice either in lysis buffer A (50 mM Tris/HCl pH 8.0, 150 mM NaCl, 1% Triton X-100, 0.01% SDS, 0.05% sodium deoxycholate) or B (50 mM Tris/HCl pH 8.0, 150 mM NaCl, 0.2% Triton X-100; for co-isolation experiments with syntenin-1/ezrin) containing 1 mM phenylmethylsulfonyl fluoride and Complete protease inhibitor cocktail (Roche, Basel, Switzerland), whereas 200,000 *g* supernatant was kept for further analysis. After centrifugation (10 min, 16,000 *g*, 4°C), the detergent lysate was divided in two fractions that were incubated with either mAb AC133–magnetic beads (45 µl/ml saliva) or goat anti-mouse IgG–magnetic beads (45 µl/ml saliva) as negative control, for 2 hours (4°C) prior to magnetic separation using MS columns. Materials retained in columns were washed with 6×1 ml of ice-cold PBS containing 2 mM EDTA and 0.1% Triton X-100, and then flushed with the same buffer. Flow-through materials were collected. Bound and unbound (flow-through) fractions and 200,000 *g* supernatant (see above) were subjected to methanol/chloroform precipitation. Proteins were resuspended in Laemmli sample buffer and analyzed by immunoblotting. Alternatively, prior to analysis, the precipitate of immuno-magnetically-isolated prominin-1 was incubated 5 hours at room temperature in the absence or presence of 1 U peptide-N-glycosidase F (PNGase F) according to the manufacturer's protocols (Roche Diagnostics GmbH, Mannheim, Germany).

### Immunoblotting

Proteins were separated by SDS-polyacrylamide-gel electrophoresis (SDS-PAGE; 6% for MUC1, 10% for CD63 and syntenin-1, 7.5% for others) and transferred to a poly (vinylidene difluoride) (PVDF) membrane (Millipore, Bedford, MA: pore size 0.45 µm) using a semi-dry transfer system (Cti, Idstein, Germany) as previously described [58]. After transfer, membranes were incubated in blocking buffer (PBS containing 5% low fat milk powder and 0.3% Tween 20) overnight at 4°C prior to being probed with primary antibody (see Table S2) for 1 hour at room temperature. Antigen-antibody complexes were detected using appropriate horseradish peroxidase (HRP)-conjugated secondary antibody (AffiniPure rabbit anti-mouse IgG, goat anti-mouse IgG2b, goat anti-mouse IgG light chain specific or goat anti-rabbit IgG; Jackson Immunoresearch) and visualized with enhanced chemiluminescence reagents (ECL system; Amersham Corp., Arlington Heights, IL). Membranes were exposed to films (Hyperfilm ECL; Amersham-Pharmacia) and quantified after scanning using ImageJ software.

## Results

### Expression of PROMININ-1 in salivary gland tumors

Paraffin-embedded sections of various lesions of human salivary glands including PA, AciCC, MEC and AdCC and obstructive SA were examined for the expression of prominin-1 using two characterized mAbs: 80B258 and AC133 [6], [19]. Our evaluation was focused on occurrence of prominin-1 immunoreactivity in relation to the cytoarchitecture including growth pattern and cytomorphology, its localization throughout the tumor and coincidence with inflammatory and hemorrhagic areas (data are summarized in Table 1).

**Table 1:** Differential immunoreactivity of prominin-1 and other cancer markers in distinct salivary gland diseases.

Case	prominin-1		CEA	MUC1	
	80B258	AC133	Parlam4	115D8	DF3
**Pleomorphic Adenoma (PA)**					
1	+	+	+	++	+
2	+	+	+	+	+
3	+	+	+	+	+
4	+	+	+	++	+
5	+	+	+	+	+
6	+	+	N.D.	N.D.	N.D.
7	+	–	N.D.	N.D.	N.D.
8	+	–	N.D.	N.D.	N.D.
9	++	+	+	+++	+
10	+	+	+	++	+
**Acinic Cell Carcinoma (AciCC)**					
1	+ [p]*	–	+	+	+
2	+ [p, h, i]	–	++	+	–
3	+ [p, h]	–	+	+	–
4	+	–	N.D.	N.D.	N.D.
5	++++	+	+	+++	+++
6	–	–	N.D.	N.D.	N.D.
7	+	–	N.D.	N.D.	N.D.
8	++	–	+	++	++
9	+ [p, i]	–	N.D.	N.D.	N.D.
10	+ [p, h]	–	+	+	+
11	+ [p, h, i]	–	+	+++	+++
**Mucoepidermoid Carcinoma (MEC)**					
1	–	–	N.D.	N.D.	N.D.
2	–	–	N.D.	N.D.	N.D.
3	–	–	N.D.	N.D.	N.D.
4	– [#]	– [#]	+	+++	+
5	+	–	+	+++	++
6	– [#]	– [#]	+	++++	++++
7	–	–	N.D.	N.D.	N.D.
8	+	–	+	+++	+
9	–	–	–	+++	++
10	–	–	N.D.	N.D.	N.D.
11	–	–	N.D.	N.D.	N.D.
12	–	–	N.D.	N.D.	N.D.
13	– [#]	– [#]	–	++++	–
14	–	–	+++	+++	+
15	– [#]	– [#]	N.D.	N.D.	N.D.
**Adenoid Cystic Carcinoma (AdCC)**					
1	+	–	+	+	+
2	++++	++	+++	+++	+++
3	++	+	N.D.	N.D.	N.D.
4	+++	++	+++	+++	+++
5	++	+	N.D.	N.D.	N.D.
6	++++	+	++	++	+
7	+++	++	N.D.	N.D.	N.D.
**Sialadenitis (SA)**					
1	+++	+	++	+++	–
2	++	+	+++	++	+
3	++	+	+++	++	–

CEA, carcinoembryonic antigen; MUC1, mucin 1.

*Occurrence: p, peripheral; h, hemorrhagic; i, inflammatory tumor areas.

# Occurrence of 80B258- and AC133-positive non-tumoral ductal structures between cancer cells.

–, negative; +, < 10%; ++, 10–25%; +++, 25–50%; ++++, >50% of tumor area.

N.D., not determined.

Note that prominin-1 immunoreactivities in peritumoral non-neoplastic tissues are not indicated.

#### Pleomorphic adenoma

**Figure 1:**
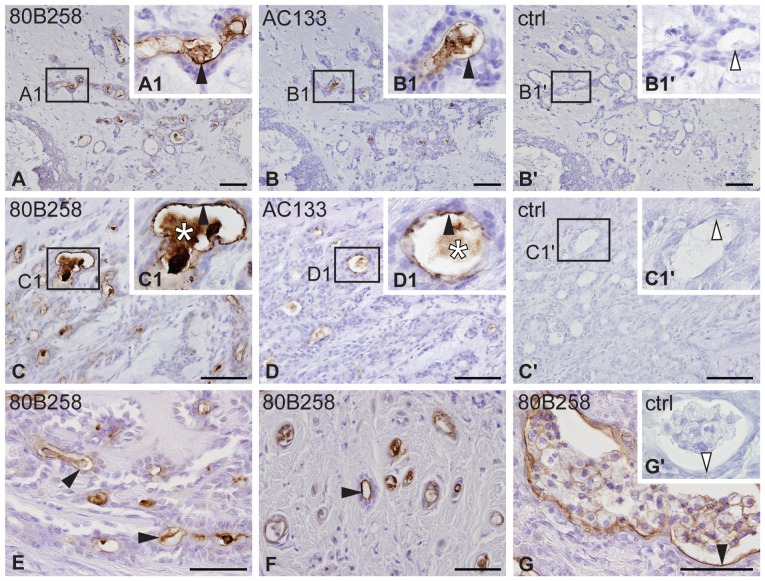
Immuno-detection of prominin-1 in pleomorphic adenoma. Adjacent sections of distinct individual PA cases were immunolabeled with either 80B258 (**A**, **C**, **E–G**) or AC133 (**B**, **D**) mAb directed against prominin-1, or isotype control (**B**′, **C**′, **G**′**,** ctrl) prior to hematoxylin counterstaining. Boxed areas (**A1–D1**) are displayed at higher magnification in the corresponding inset. Filled black and hollow arrowheads indicate the presence (A**–**G) or absence (B′, C′, G′) of prominin-1 at the apical membrane of cells of rare duct-like structures, respectively. Asterisks indicate the immune–positive secretion. Histopathological characteristics of individual cases: #1 (A, B), #2 (C, D) and #5 (E**–**G) are summarized in Table S1. Scale bars 50 µm.

In PA of parotid glands, 80B258 immunoreactivity was observed at the apical plasma membrane of epithelial cells of rare duct-like structures (Fig. 1A, C, E–G, black arrowhead), and in their secretion (Fig. 1C, asterisk). Only a subset of them exhibited a weak AC133 immunoreactivity (Fig. 1B, D). 80B258–positive structures were observed throughout the whole tumor with an overall occurrence representing less than 10% of the area (Table 1). Chondroid, myxochondroid and hyalinized areas that constitute mesenchymal-like component were negative (Fig. S1A–C). No immunoreactivity was observed with the isotype control (Fig. 1B′, C′, G′, ctrl).

#### Acinic cell carcinoma

**Figure 2:**
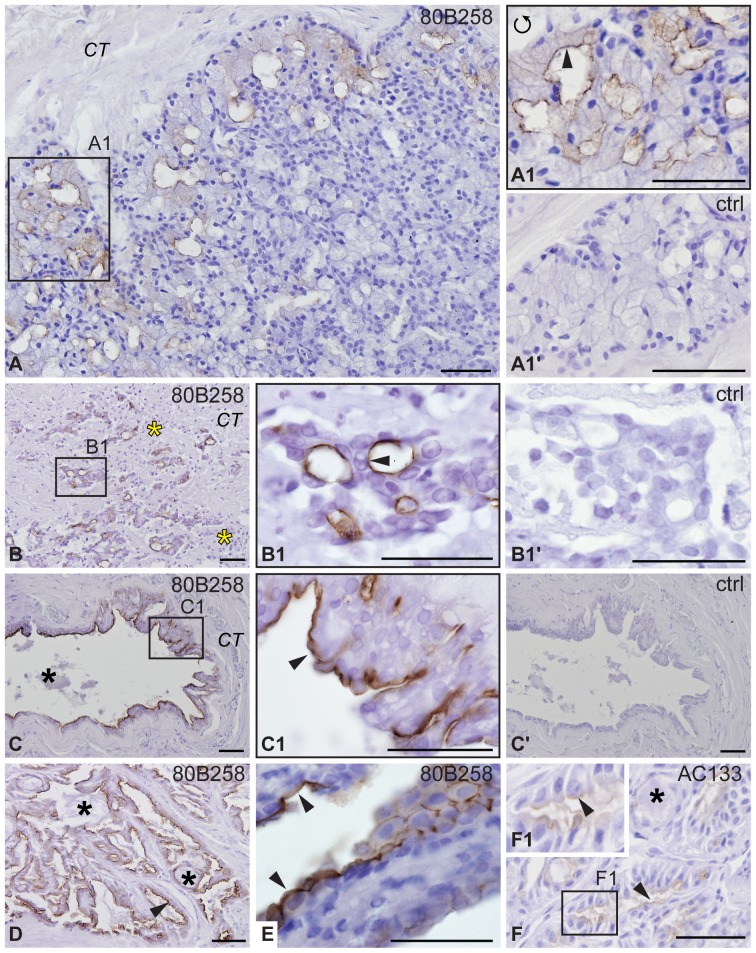
Immuno-detection of prominin-1 in acinic cell carcinoma. Adjacent sections of distinct individual AciCC cases were immunolabeled with either 80B258 (**A–E**) or AC133 (**F**) mAb directed against prominin-1, or isotype control (**A1**′, **B1**′**,** **C**′, ctrl) prior to hematoxylin counterstaining. Boxed areas (**A1–C1**, **F1**) are displayed at higher magnification. Black arrowheads indicate prominin-1 at the apical membrane of acinar (A1) and intercalated duct-like (B1, C1–F1) cells in tumors with either microcystic (A, B) or papillary cystic (C-F) growth patterns. Black and yellow asterisks show the negativity of secretion and leukocyte infiltration, respectively. *CT*, connective tissue surrounding tumor. Histopathological characteristics of individual cases: #1 (A), #2 (B), #4 (C), #5 (D, F) and #8 (E) are summarized in Table S1. Scale bars 50 µm.

In AciCC, 80B258 immunoreactivity was observed in regions with microcystic (MC; Fig. 2A, B) and papillary-cystic (PC; Fig. 2C–E) growth patterns, where it constituted less than 10% and more than 25% of the total areas, respectively (Table 1). In MC growth pattern, 80B258–positive cells were predominantly found in the peripheral parts (6 of 11 cases (6/11)) of either lobularly-growing tumor (Fig. 2B) or individual nodules (Figs. 2A, S2A, B), and/or in hemorrhagic (Fig. S2C, red asterisk; 4/11) or inflammatory areas (Fig. 2B, yellow asterisk; 3/11). In general, 80B258 immunoreactivity was detected at the apical plasma membrane of tumor cells with acinar (Fig. 2A1, arrowhead) or intercalated duct-like (Fig. 2B1, arrowhead) morphology. In PC growth pattern, 80B258 immunoreactivity was observed in intercalated duct-like cells distributed throughout the entire tumor (Fig. 2C1, D, E, arrowhead). In contrast, AC133 immunoreactivity was detected only sporadically at the apical membrane of some tumor cells with PC growth pattern (Fig. 2F, arrowhead). Secretion present in the lumina usually lacked prominin-1 immunoreactivity (Fig. 2C, D, F, asterisk).

The acinar (Fig. S1D1, E1, black arrow) or non-specific glandular (Fig. S1E2, white arrow) cells found in regions with solid growth pattern were negative. No immunoreactivity was detected when using an isotype control (Fig. 2A1′, B1′ C′, ctrl).

#### Mucoepidermoid carcinoma

**Figure 3:**
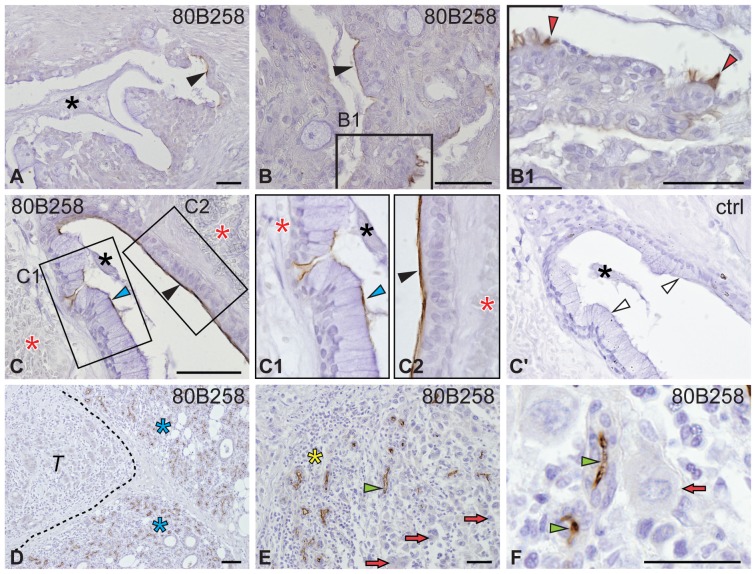
Immuno-detection of prominin-1 in mucoepidermoid carcinoma. Sections of distinct individual MEC cases were immunolabeled with either 80B258 mAb (**A–F**) directed against prominin-1 or isotype control (**C**′, ctrl) prior to hematoxylin counterstaining. Boxed areas (**B1**, **C1**, **C2**) are displayed at higher magnification. Black, blue and red arrowheads point to the 80B258 immunoreactivity at the apical membrane of some columnar (A, B, C, C2), mucous (C, C1) and ciliated (B1) cells lining cystic tumor structures, respectively. Red arrows indicate the negativity of anaplastic cells (E, F). Dashed line demarcates tumor (*T*) from surrounding normal tissue (blue asterisk, D). Black asterisk, immunonegative secretion; red asterisk, hemorrhagic regions; yellow asterisk, leukocyte infiltration; green arrowhead, non-cancerous intercalated ducts. Histopathological characteristics of individual cases: #8 (A), #5 (B, C) and #13 (D–F) are summarized in Table S1. Scale bars 50 µm.

In MEC, 80B258 immunoreactivity was observed solely in two of fifteen cases, displaying a low/intermediate-grade and cystic or papillary-cystic cell arrangement (Table 1). Therein, the immunolabeling was found in some cyst-like structures and confined to the apical plasma membrane of either non-characteristic columnar cells (Fig. 3A–C, C2, black arrowhead) or cells with evident mucous cell differentiation, i.e. with a flattened nucleus located at the basal portion and cytoplasm filled with mucin granules (Fig. 3C, C1, blue arrowhead). Moreover, cilia of rare differentiated tumor cells were positive (Fig. 3B1, red arrowhead). A coincidence with hemorrhage in the vicinity of 80B258–positive structures was observed in one case (Fig. 3C, red asterisk). Immunoreactive structures covered less than 10% of the tumor area (Table 1). Secretion (Fig. 3A, C, black asterisk) as well as intermediate (Fig. S1F, white arrow), squamous (Fig. S1G, H, blue arrow), clear (Fig. S1I–K, black arrow) and anaplastic cells (Fig. 3E, F, red arrow) were negative. No immunoreactivity was detected using AC133 mAb (Table 1). Interestingly, no prominin-1 immunoreactivity was detected in high-grade carcinomas (Table 1). In those with unequivocal infiltrative growth, we could nevertheless observe 80B258/AC133-immunoreactive remnants of intercalated ducts of original salivary gland tissue (Fig. 3D–F, green arrowhead; Table 1), often with abundant leukocyte infiltration interspersed among tumor tissues (Fig. 3E, yellow asterisk). In all situations, no immunoreactivity was detected using the isotype control (Fig. 3C′, white arrowhead, ctrl).

#### Adenoid cystic carcinoma

**Figure 4:**
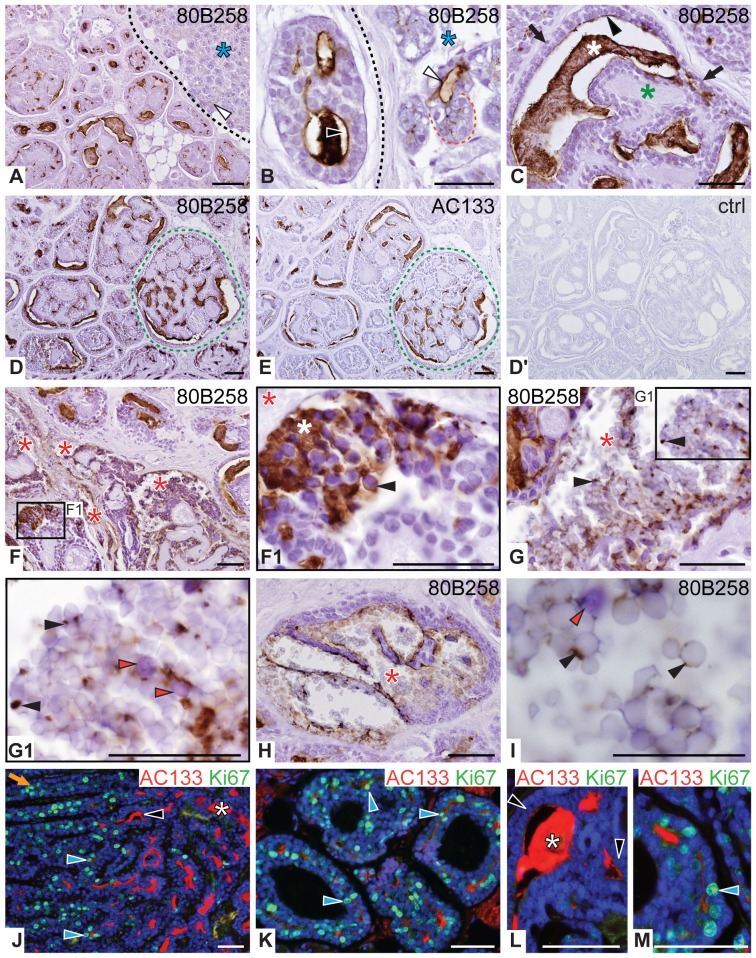
Immuno-detection of prominin-1 in adenoid cystic carcinoma. Adjacent sections of AdCC with cribriform growth pattern were immunolabeled with either 80B258 (**A–D**, **F–I**) or AC133 (**E**) mAb directed against prominin-1, or isotype control (**D**′, ctrl) prior to hematoxylin counterstaining. Alternatively, sections were subjected to double immunofluorescent labeling with AC133 mAb (red) and Ki67 antiserum (green). Nuclei were visualized with DAPI (**J–M**). Boxed areas (**F1**, **G1**) are displayed at higher magnification. Green dashed lines indicate corresponding structures on consecutive sections. Non-cancerous surrounding tissue (A, B, blue asterisk) is delimited from tumor by black dashed line; red dotted line indicates serous acinus. Filled black and white arrowheads point to prominin-1 at the apical membrane lining epithelia of tumor duct-like structures (C, J, L) or non-cancerous intercalated ducts (A, B). Black arrowheads indicate prominin-1 within the cytoplasm and/or entire cell membrane of cancer cells (F1) or in secreted materials mixed with erythrocytes (G, I) present in hemorrhagic areas (F–I, red asterisk). Red arrowheads label free (i.e. non-attached) tumor cells between erythrocytes (G1, I). White asterisk indicates prominin-1 immunoreactive secretion in true lumina (C, F1, L) whereas green asterisk and black arrow show pseudocysts and basaloid myoepithelial cells (C) lacking prominin-1, respectively. Blue arrowheads and orange arrows (J–M) indicate Ki67–positive cells that co-express or not AC133, respectively. Histopathological characteristics of the displayed case #2 (A-M) are summarized in Table S1. Scale bars 50 µm.

In AdCC, 80B258–positive structures were observed throughout the whole tumor and covered 10–75% of the total areas (Fig. 4; Table 1), which strikingly contrasts with the other types of carcinomas investigated. Interestingly, most of duct-like structures characteristic of cribriform growth pattern, which was predominantly present in our samples, displayed the 80B258 immunoreactivity (Fig. 4A, D). Specifically, 80B258 immunoreactivity was confined to the apical plasma membrane of intercalated duct-like cells present within the tumor (Fig. 4B, C, black arrowhead) and its non-neoplastic vicinity (Fig. 4A, B, white arrowhead). Basaloid myoepithelial cells and pseudocysts composed of mucopolysaccharide-rich stroma of tumor were negative (Fig. 4C, black arrows and green asterisk, respectively). Remarkably, intense 80B258 immunoreactivity was detected in the secretion present in the lumen of duct-like structures (Fig. 4C, white asterisk). Although generally weaker, the AC133 labeling gave a similar outcome in both ductal structures and secretion (Fig. 4E). In 3 of 7 cases, it is of note that we observed the 80B258 immunoreactivity within the cytoplasm and/or the entire plasma membrane of cells in either solidly growing structures, which contain non-luminal cells (Fig. S3B–D, blue arrowhead), or duct-like structures comprising usually delaminated cells surrounded by abundant secretion (Fig. 4F1, black arrowhead and white asterisk, respectively). Often, structures that contain cells with an unpolarized distribution of prominin-1 immunoreactivity were found in the vicinity of numerous intact or disrupted thin-walled blood vessels (Fig. 4F–H, red asterisk) suggesting a potential communication of erythrocytes with tumor cells and/or secreted materials (Fig 4G–I, S3C, red and blue arrowheads). No immunoreactivity was detected using isotype control (Figs. 4D′, S3A–F, ctrl).

To find out whether prominin-1–positive cells are confined to actively proliferating ones we performed double immunofluorescence labeling with the proliferating cell marker Ki67. Interestingly, Ki67–positive cycling cells predominated in regions with AC133 immunonegative tumor cells (Fig. 4J, orange arrow) or were present in newly generated and narrow duct-like structures that are positive for AC133 (Fig. 4J, K, M, blue arrowhead). In contrast, larger lumina with copious AC133 immunoreactive secretions (Fig. 4J, L, white asterisk) were surrounded by non-proliferating AC133–positive cells (Fig. 4L, black arrowhead).

### Increase of PROMININ-1 expression in peritumoral areas and in sialadenitis

**Figure 5:**
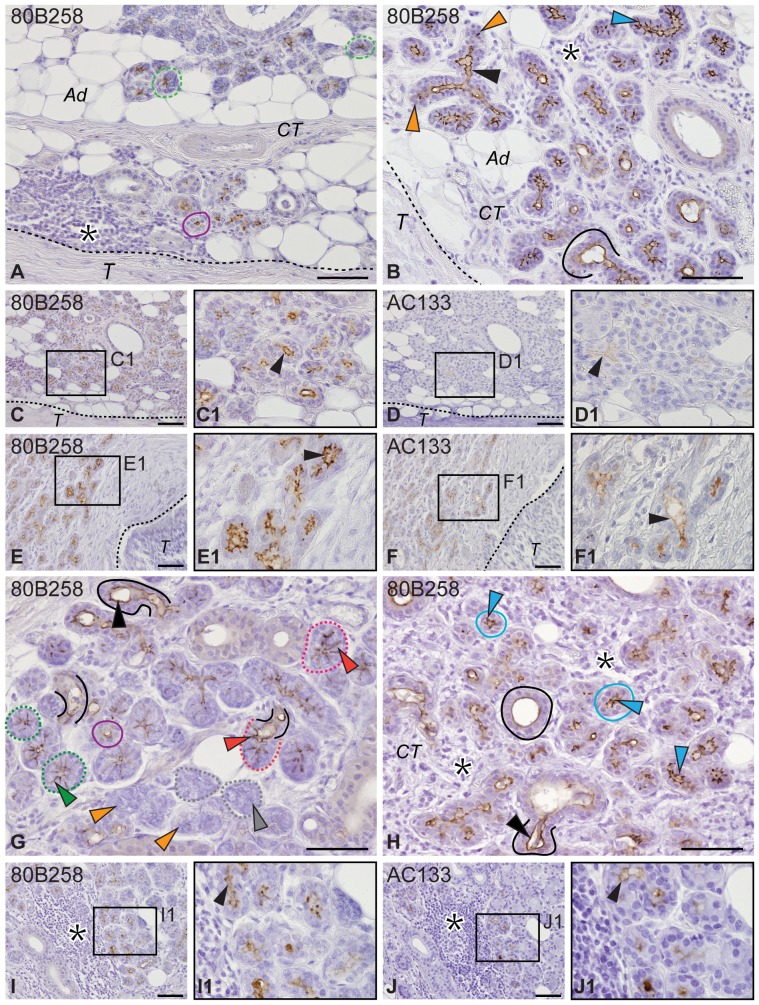
Immuno-detection of prominin-1 in tumor-surrounding tissues and sialadenitis. Sections of non-neoplastic peritumoral tissues of PA (**A**, **B**, **C**, **D**), MEC (**E**, **F**) and SA from obstructive reasons of early (**G**, **I**, **J**) and later (**H**) stages, respectively, were immunolabeled with 80B258 (A–C, E, G–I) or AC133 (D, F, J) mAb directed against prominin-1 prior to hematoxylin counterstaining. Sets of C/D, and E/F and I/J images show corresponding areas in consecutive sections. Boxed areas (**C1–F1**, **I1**, **J1**) are displayed at higher magnification. Non-neoplastic tissue is delimited from tumor (*T*; A–F) by dashed line. Black asterisks indicate inflammatory regions (A, B, H–J). Green, red and grey dashed lines indicate normal (A, G), degenerating (G) or atrophying (G) acini containing narrow, dilated or non-recognizable intercellular canaliculi (G, labeled with corresponding color arrowhead), exhibiting weak, strong or no 80B258 immunoreactivity, respectively. Orange arrowheads show atrophic secretory endpieces (B, G). Violet, black and blue lines indicate normal (A, G), dilated (B, G, H) and newly formed (H) intercalated ducts, respectively, with smooth or in latter case irregular lumen. Black and blue arrowheads indicate apical (B, C1–F1, G, H, I1, J1) and apicolateral (B, H) cell membrane immunoreactivity with 80B258 (B, C1, E1, G, H, I1) or AC133 (D1, F1, J1) mAb. *Ad*, adipocytes, *CT*, connective tissue. Histopathological characteristics of individual cases of PA: #9 (A) and #5 (B, C, D), MEC: #4 (E, F), and SA: #2 (G, I, J) and #1 (H) are summarized in Table S1. Scale bars 50 µm.

In the non-neoplastic salivary gland tissues that surrounded the tumors (Fig. 5A–F; *T*), and in cases of obstructive SA (Fig. 5G–J), we observed an enhanced prominin-1 expression in intercalated ducts using both 80B258 and AC133 mAbs (Fig. 5C–F, I, J, black arrowhead). AC133 exhibited a weaker reactivity by comparison to 80B258, but was nevertheless regularly detected. In both conditions, we found various degrees of inflammation with periductal leukocyte infiltration (Fig. 5A, B, H–J, black asterisk) and atrophy of functional secretory tissues (Fig. 5B, G, orange arrowhead) leading to their replacement by intercalated duct-like cells (Fig. 5B, H) and/or adipose tissue (Fig. 5A, B, *Ad*) or connective tissue (Fig. 5A, B, H, *CT*). Variations of prominin-1 immunoreactivity within the acini were associated with their complex alteration. First, the degeneration of acinar cells where prominin-1 is normally weakly expressed (Fig. 5A, G, green dashed line and arrowhead) led to its complete absence therein (Fig. 5G, grey dotted line and arrowhead). Second, saliva stasis caused dilation of intercellular canaliculi between serous acinar cells (Fig. 5G, red arrowhead), which provoked their atrophy (Fig. 5B, G, orange arrowhead) and/or their replacement by proliferating intercalated duct cells (i.e. cells without basophilic secretory granules). This resulted in an intense prominin-1 labeling either at the apical (Fig. 5B, black arrowhead) or apicolateral (Fig. 5B, H, blue arrowhead) membrane of newly formed duct-like cells. Third, normal intercalated ducts with small and regular lumen (Fig. 5A, G, violet line) became dilated with or without an irregular lumen (Fig. 5B, G, H, black line) and gave rise to a strong prominin-1 expression in luminal cells (Fig. 5G, H, black arrowhead).

### Partial co-expression of PROMININ-1 with CEA and MUC1

To find out whether a relation between the expression of prominin-1 and other cancer markers such as CEA and MUC1 exists, we re-examined our samples with specific and characterized antibodies. The Parlam 4 mAb specifically labels CEA without cross-immunoreactivity with other CEA relative proteins [55], whereas 115D8 [57] and DF3 [35] mAbs are directed against carbohydrate and core peptide epitopes of MUC1, respectively [59]. The latter antibodies are employed as catcher and tracer in commercially available cancer immunoassays for CA15-3 [60].

**Figure 6:**
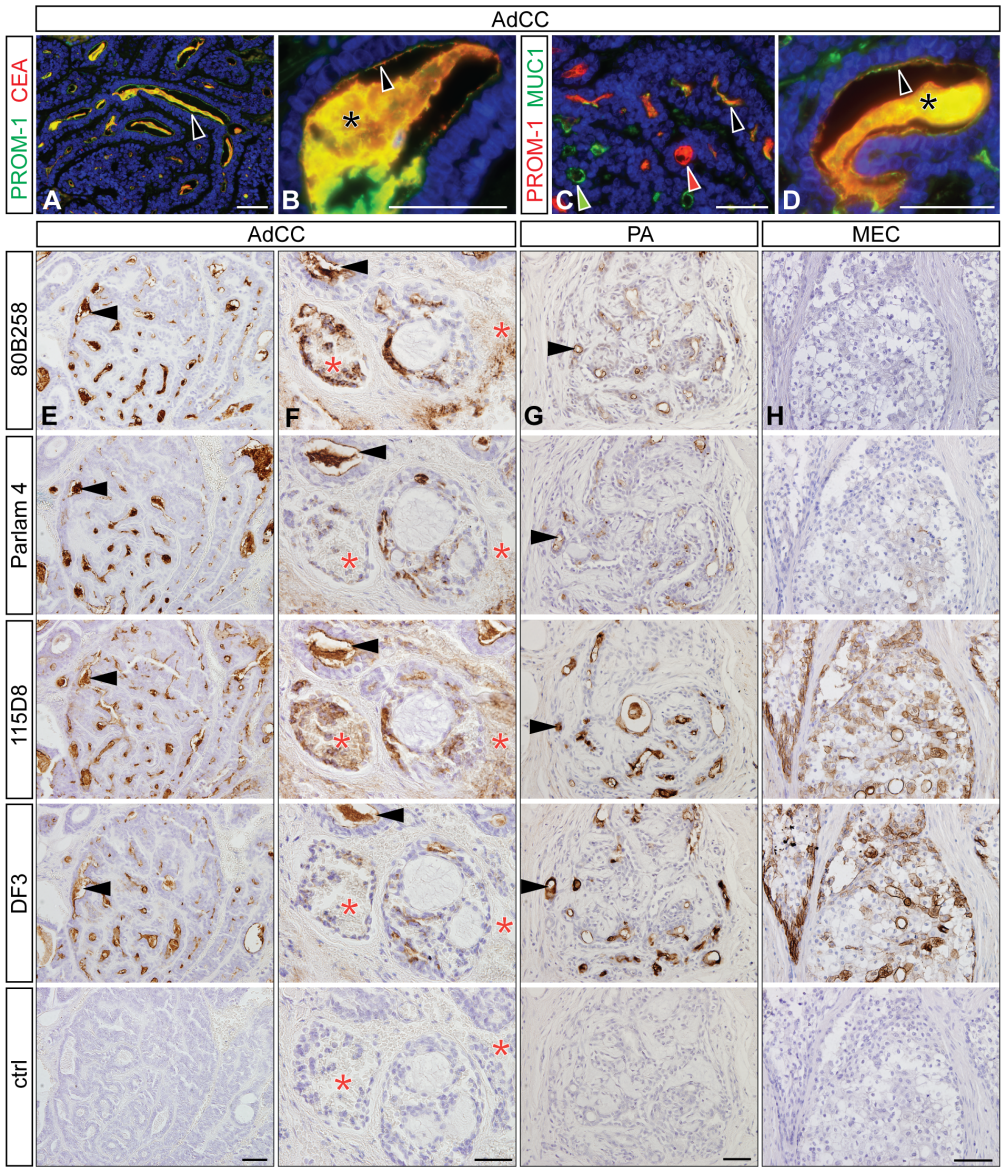
Partial co-expression of prominin-1 with CEA and MUC1 in various salivary gland tumors. AdCC samples (**A–D**) were double immunolabeled with anti-prominin-1 mAb 80B258 (PROM-1; A**–**D) and either anti-CEA antiserum (CEA, A, B) or anti-MUC1 mAb 115D8 (MUC1, C, D) followed by the appropriate fluorochrome-conjugated secondary antibody. Nuclei were visualized with DAPI. Co-expression of prominin-1 with CEA or MUC1 at the apical membrane (B, D, black arrowhead) and secretion (asterisk) are indicated. Arrowheads (C) indicate prominin-1 (red) or MUC1 (green) non-overlapping and overlapping (black) immunoreactivities in duct-like structures. Alternatively, consecutive sections of AdCC (**E**, **F**), PA (**G**) and MEC (**H**) were peroxidase-immunolabeled using mAbs 80B258, Parlam 4 and 115D8/DF3 directed against prominin-1, CEA and MUC1, respectively (E–H) or isotype control (E–H, ctrl) prior to hematoxylin counterstaining. Note the similar expression pattern in corresponding structures (black arrowheads) in AdCC and PA, but not in MEC. Red asterisk, hemorrhagic region. Histopathological characteristics of individual cases of AdCC: #2 (A–F), PA: #5 (G) and MEC: #5 (H) are summarized in Table S1. Scale bars 50 µm.

In AdCC, where a strong expression of prominin-1 was found, we observed co-expression with CEA either at the apical plasma membrane of cells forming the majority of duct-like structures (Fig. 6A, B, arrowhead) or in secretion (Fig. 6B, asterisk). In the case of MUC1, some structures were co-expressing both markers (Fig. 6C, D, black arrowhead) while others expressed only one or the other (Fig. 6C, red and green arrowheads). Additional cases of AdCC confirmed that the expression pattern of prominin-1 corresponds to that of both CEA and MUC1 in terms of structures (ductal structures, secretion, Figs. 6E, S3A, arrowhead), subcellular localization (apical membrane, Figs. 6F, S3B, black arrowhead; whole membrane immunoreactivity, Fig. S3B–D, blue arrowhead), and site localization (hemorrhagic regions, Figs. 6F, S3B–D, red asterisk). However, all antibodies did not always provide completely overlapping signals (Fig. S3B–D, see the weaker DF3 immunoreactivities by comparison to the 115D8 ones; Fig. S3E, F, see alternating CEA or MUC1 immunoreactions, black arrowhead). A similar co-expression pattern was observed for PA (Figs. 6G, S4A, B) and AciCC samples (Fig. S2A–D). Therein, immunoreactivities were present at the apical membrane of luminal cells found in cystic/ductal structures (Figs. 6G, S4A, B, S2B1, C1, black arrowhead) as well as in hemorrhagic (Fig. S2C, red asterisk) or peripheral neoplastic regions (Fig. S2A–C). However, the extent of the CEA- and MUC1-immunoreactive areas was greater than that of prominin-1 (Table 1). Occasionally, we observed prominin-1–positive structures co-expressing either CEA or MUC1 (Fig. S2A, D, black arrowhead).

Substantial differences were observed in MEC. In contrast to the strong expression of MUC1, prominin-1 and CEA immunoreactivities were only rarely present (Fig. S5A, F, orange and green arrowheads, respectively) or even absent (Figs. 6H, S5B–E). The expression of MUC1 appeared at the plasma membrane and in the cytoplasm of the majority of tumor cell types (Figs. 6H, S5A–F, Table 1).

Finally, the peritumoral non-neoplastic tissues and SA cases showed co-expression of prominin-1, CEA and MUC1 (115D8) at the apical membrane of secretory and duct epithelia (Fig. S4C, D and E, F, black arrowhead). The detection of MUC1 with DF3 mAb was usually weaker (Fig. S4D) and/or rarely present (Fig. S4D–F). No immunoreactivity was detected using isotype controls (Figs. 6, S2–S5, ctrl).

### PROMININ-1, CEA and MUC1 are associated with membrane vesicles

The co-expression of prominin-1 with CEA and MUC1 in the examined tissues led us to investigate whether the latter two proteins were also associated with the prominin-1–containing membrane vesicles found in saliva [21]. Such particles can be recovered either upon ultracentrifugation at 200,000 *g* [21], [29] or isolated with AC133 mAb-conjugated to magnetic beads [28].

**Figure 7:**
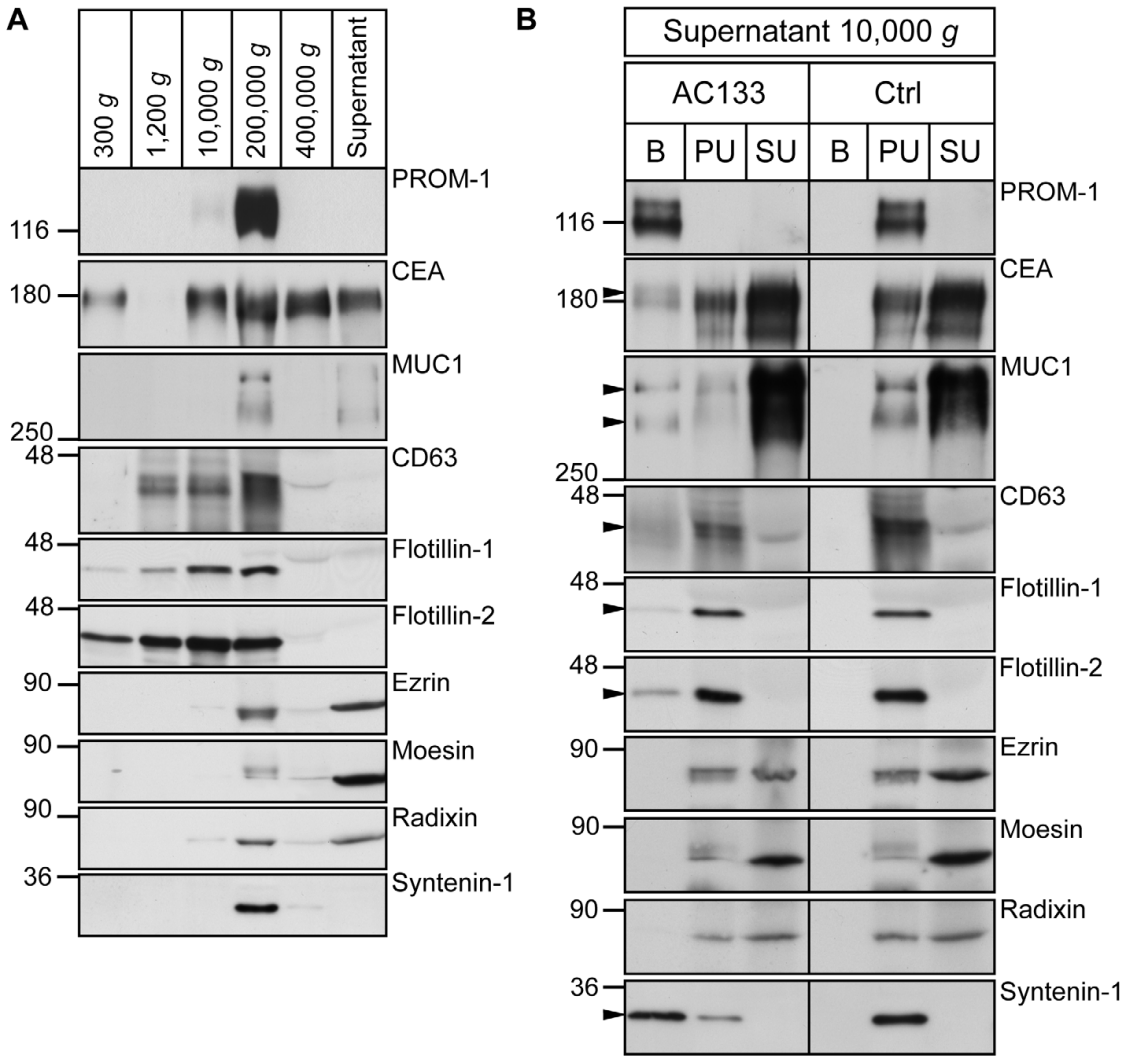
Association of CEA, MUC1, CD63, flotillin-1/2 and syntenin-1 with saliva-derived prominin-1–containing membrane vesicles. (A) Human saliva was subjected to differential centrifugation for 5 min at 300 *g*, 20 min at 1,200 *g*, 3 min at 10,000 *g*, 1 h at 200,000 *g* and 1 h at 400,000 *g*. Proteins in the 400,000 *g* supernatant were recovered by precipitation. Pellets were analyzed by immunoblotting using antibodies against various markers as indicated. (B) Membrane vesicles found in the 10,000 *g* supernatant was subjected to immuno-isolation using AC133 mAb (AC133) or goat anti-mouse Ab as a control (Ctrl). Bound (B) and unbound (PU) fractions were centrifuged at 200,000 *g* and both pellets together with the supernatant of the unbound fraction (SU) recovered by precipitation were analyzed using specific antibodies. prominin-1 was detected using 80B258 (A) or C24B9 (B) mAb. Arrowheads indicate corresponding proteins present in prominin-1–positive vesicles. Aliquots corresponding to 500 µl of saliva were loaded. Molecular mass markers (kDa) are indicated.

Differential centrifugation of human saliva followed by immunoblotting revealed that CEA and MUC1 were sedimented in the 200,000 *g* pellet fraction as prominin-1 (Fig. 7A). CEA and MUC1 were also detected in other fractions indicating either their association with other membranous components (10,000 *g* and 400,000 *g* pellets) or the presence of soluble forms (400,000 *g* supernatant). Other membranous (e.g., CD63, flotillin-1 and flotillin-2) and cytoplasmic (e.g., syntenin-1) proteins previously reported to be associated with exosomes (i.e. small membrane vesicles released from multi-vesicular bodies) [61] were recovered in the 200,000 *g* pellet fraction as well (Fig. 7A). The adaptor proteins of the ezrin/radixin/moesin (ERM) family were also found in this fraction and in the supernatant (Fig. 7A). Next, we immuno-isolated prominin-1–containing membrane vesicles using magnetic-bead technology with mAb AC133 and probed them by immunoblotting. Interestingly, we observed that CEA and MUC1 were partly associated with these vesicles (Fig. 7B, bound (B) fraction), which also contained CD63, flotillin-1, flotillin-2 and syntenin-1. ERM proteins were not found therein (Fig. 7B). Quantification showed that the ratio of individual molecules associated with prominin-1–positive vesicles represented 9.6±6.7% (CEA; mean ± standard deviation, n = 3–4), 38±5.4% (MUC1), 27.5±13.6% (CD63), 6.7±2.8% (flotillin-1), 8.4±3.5% (flotillin-2) and 78 17% (syntenin-1) of the total immunoreactive proteins present in materials recovered upon 200,000* g* centrifugation (Fig. 7B, bound (B) + unbound flow-through (PU)).

### PROMININ-1 is subjected to ubiquitination

**Figure 8:**
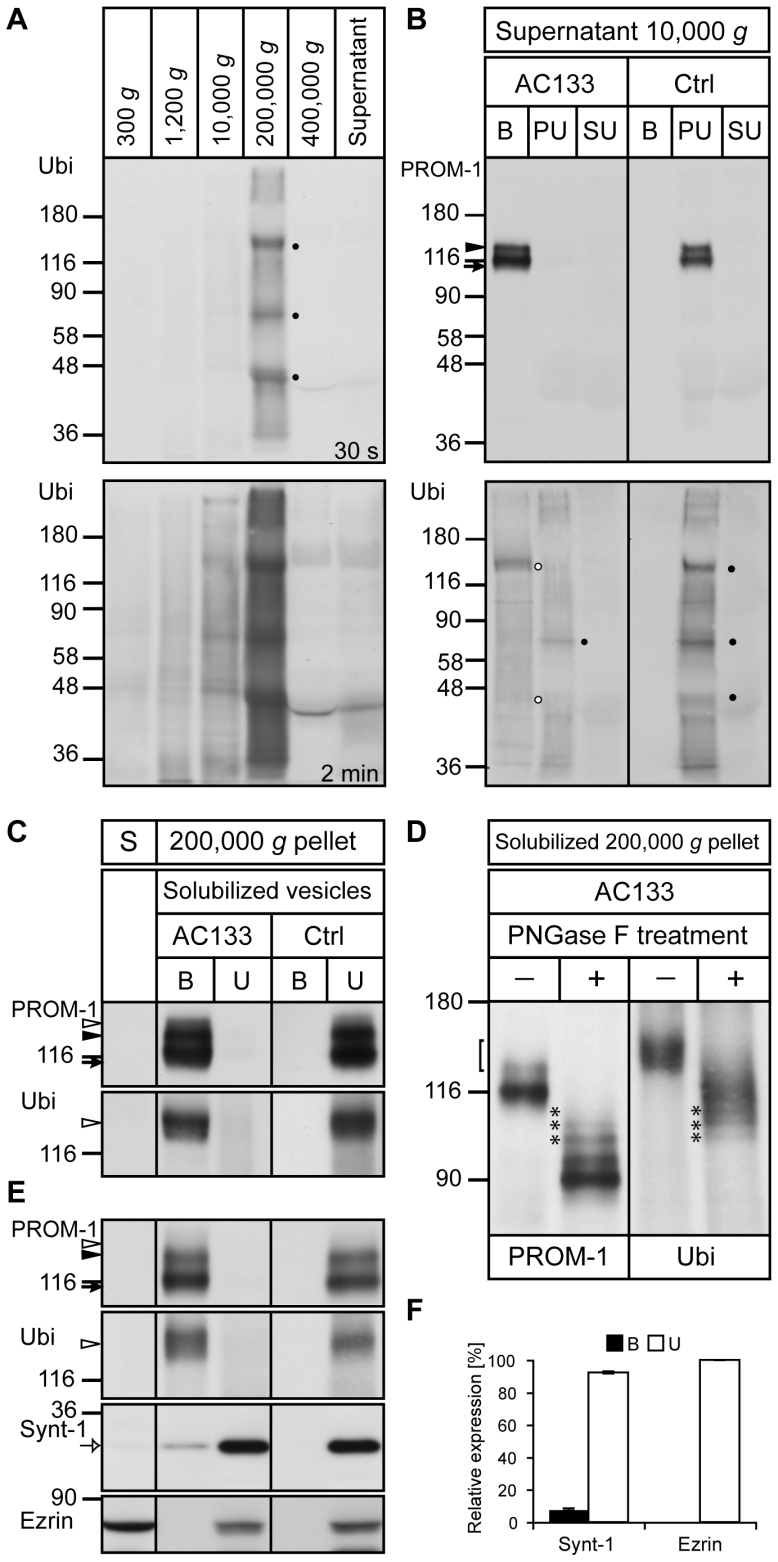
prominin-1 is ubiquitinated and interacts with syntenin-1. (**A**) Human saliva was subjected to differential centrifugation and the resulting fractions were analyzed by immunoblotting using anti-ubiquitin Ab (Ubi). Major ubiquitinated proteins recovered in the 200,000 *g* pellet are indicated (dots). A longer exposure time revealed numerous ubiquitinated proteins. (**B**) Membrane vesicles found in the 10,000 *g* supernatant were subjected to immuno-isolation using AC133 mAb (AC133) or goat anti-mouse Ab as a control (Ctrl). Bound (B) and unbound (PU) fractions were pelleted by centrifugation whereas supernatant of unbound (SU) fraction was precipitated. Pellets were analyzed using prominin-1 (C24B9) or ubiquitin (Ubi) antibodies. Major ubiquitinated proteins recovered in the bound fraction (AC133) are indicated (white dots). (**C–F**) Saliva was subjected to centrifugation and 200,000 *g* pellet was solubilized in buffer A (C, D) or B (E), whereas proteins in the supernatant (S) were recovered by precipitation (C, E). prominin-1 in the detergent lysates was subjected to immuno-isolation. Bound (B) and unbound (U) materials were analyzed for prominin-1, ubiquitin, syntenin-1 (Synt-1) and ezrin. Alternatively, AC133-bound materials were subjected to a PNGase F treatment (D). Arrow and black arrowhead indicate major prominin-1 bands while open arrowhead points the ubiquitinated prominin-1 (C, E). Bracket and asterisks indicate the upper glycosylated/deglycosylated ubiquitinated prominin-1 (D). Open arrow shows syntenin-1 co-immunoprecipitated with prominin-1 (E). The amount of syntenin-1 and ezrin associated with prominin-1 was quantified (F). Aliquots corresponding to 500 (A–C, E) and 1,250 (D) µl of saliva were loaded. Molecular mass markers (kDa) are indicated.

To gain more insights into the materials recovered upon high centrifugation, we performed immunoblotting for ubiquitin on each fraction obtained after the differential centrifugation of saliva. Interestingly, the 200,000 *g* pellet fraction contained a set of ubiquitinated proteins with three unidentified major ones (Fig. 8A, top panel, dots). To assess their association with prominin-1–containing membrane vesicles we performed immuno-isolation of these vesicles using mAb AC133–magnetic beads as above (Fig. 8B, top panel). Immunoblotting of the bound (B) and unbound (pellet (PU) and supernatant (SU)) fractions revealed that indeed part of the ubiquitinated proteins was associated with prominin-1–positive vesicles (Fig. 8B, bottom panel, white dots). Surprisingly, one of the two major ubiquitinated proteins showed the same electrophoretic mobility as the upper prominin-1-immunoreactive species suggesting that prominin-1 itself might be ubiquitinated (Fig. 8B, top panel, arrowhead). Solubilization of membrane materials recovered in the 200,000 *g* pellet prior to immuno-isolation of prominin-1 and probing with anti-ubiquitin indicated that the upper smear-like fraction of prominin-1 species was indeed ubiquitinated (Fig. 8C, open arrowhead). Deglycosylation of prominin-1 using PNGase F revealed the presence of multiple immunoreactive bands for both prominin-1 and ubiquitin suggesting that prominin-1 is mono and multi-ubiquitinated (Fig. 8D, asterisks). Not all prominin-1 present in membrane vesicles are ubiquitinated given that high-molecular-weight species of glycosylated/deglycosylated prominin-1 represent only a minor fraction of prominin-1 immunoreactivity (Fig. 8D, bracket and asterisks, respectively).

### PROMININ-1 interacts with syntenin-1

The high ratio of syntenin-1 in prominin-1–containing membrane vesicles prompted us to evaluate whether this adaptor protein interacts with prominin-1. We observed that upon solubilization of membrane materials recovered in 200,000 *g* pellet using 0.2% Triton X-100 (or 0.5%, data not shown), a fraction of the syntenin-1 (7.76±0.74%, n = 3) remained associated with immuno-isolated prominin-1 while ezrin did not (Fig. 8E, open arrow, Fig. 8F).

## Discussion

The present study highlights three major features of prominin-1. First, it reveals new insights into the histological expression of prominin-1 in human salivary gland lesions. Second, it demonstrates the ubiquitination of this molecule. Third, it identifies a novel prominin-1 interacting partner, namely syntenin-1.

The general interest of prominin-1 has grown exponentially since this cell surface molecule, and particularly its AC133 epitope, marks cells harboring stem and cancer stem cell properties. Others and we have nevertheless raised concerns about data solely acquired with the mAb AC133 since the corresponding epitope could be either down regulated or masked under certain circumstances [17], [19], [62](reviewed in [63], [64]). As a net result, the overall expression of prominin-1 protein *per se* appears to be more widespread than the AC133 antigen in healthy and cancerous tissues as previously illustrated in normal salivary glands [19]. A similar phenomenon was observed for tumor tissues and inflammatory regions of salivary glands in the present study using two anti-prominin-1 (AC133 and 80B258) antibodies on consecutive sections (Table 1). Irrespective of the tissue conditions, AC133 immunoreactivity was always weaker and/or present only in a subpopulation of 80B258–positive cells that are found in intercalated duct-like structures, and was absent in secretory (mucus and serous acinar) cells, which is in line with the proposed location of stem and progenitor cells in the intercalated ducts [65]. The robust expression of AC133 antigen noted in cells within AdCC as well as in secretion might then reflect the aberrant proliferation/differentiation of stem and progenitor cells. Coincidently, all prominin-1–containing membrane vesicles acquired from saliva of healthy donors were isolated using mAb AC133. A similar phenomenon is also observed for prominin-1–containing membrane vesicles found in the urine (J.K. and D.C., unpublished data). Therefore, unless the AC133 epitope gains a better accessibility in highly curved membrane of small vesicles, this tends to suggest that such vesicles might originate from intercalated duct cells and not acinar serous and mucous cells, where prominin-1 is detected solely with mAb 80B258.

Regarding tumor tissues, we found that prominin-1 was expressed extensively in AdCC, to a lesser extent in AciCC and PA and only in scattered cells in MEC. Generally, its presence was detected in duct- and cyst-like structures, which are mainly characteristic of adenomas and low-grade carcinomas, i.e. tumors that maintain certain differentiated tissue morphology. Less differentiated cancer tissues were negative for prominin-1 as observed particularly in high-grade MEC. These findings are in agreement with prominin-1 expression in other epithelial cancer tissues including pancreatic adenocarcinoma, cholangiocarcinoma and colorectal carcinoma [62], [66], [67]. Although the tumor cases mentioned in these studies had in common a well/moderately differentiated morphology, some were surprisingly connected with a worse prognosis than those poorly differentiated with a prominin-1–negative phenotype, which indicates that the detection of prominin-1 might be a useful tool for diagnosis, and eventually, for the choice of adequate treatment [66].

The subcellular localization of prominin-1 might also be instructive about the origin and progression of cancers. Irrespective of the tumor types, prominin-1 was often detected at the apical membrane of polarized tumor cells that resembled secretory serous and mucous cells as well as intercalated duct cells of normal salivary glands [19]. Thus, its expression seems to be preserved in tumor cells that originate from prominin-1–positive cells. We also, yet rarely, observed that prominin-1 was distributed throughout the entire plasma membrane and/or cytoplasm of cells in AdCC, suggesting a loss of cell polarization and cell-cell contact. Such tumor cells were often found in the vicinity of thin-walled blood vessels, that highlights a potential communication between them. The presence of prominin-1 in extracellular membrane vesicles that are used as a communication device (see below) is consistent with such hypothesis. It remains to be clarified whether any relation between the non-polarized expression of prominin-1 by cancer cells and the pathological neovascularization exists [16]. The lack of prominin-1 polarization could also reflect an epithelial mesenchymal transition [68] that eventually results in metastasis [69], [70]. In this context, it will be interesting to determine if the number of prominin-1–positive cells is increasing in bloodstream as previously demonstrated in breast carcinoma and non-small cell lung cancer patients [71]–[73].

Intriguingly, we observed an enhanced expression of prominin-1 in intercalated ducts within inflammatory regions found in SA and peritumoral non-neoplastic tissues of salivary glands. Similar observations were made in kidney, exocrine pancreas and prostate cancers [17], [20], [62]. In prostate, prominin-1–positive cells exhibited a shrunken morphology characteristic of proliferative inflammatory atrophy [20]. In salivary gland tissues, cellular alterations were represented by an atrophy of secretory components and an expansion of prominin-1–positive intercalated duct cells. These histopathological changes in peritumoral tissues might be the consequence of chronic irritation provoked by the expansion of tumor tissue leading to possible compression, blood circulation stasis and hypoxia. The inflammation could predispose tissues to cancer development. Indeed, extensive expression of prominin-1 in epithelial cancer cells was demonstrated in a murine model of chronic intestinal inflammation that develops spontaneously adenocarcinomas [74]. Another indicator illustrating a certain link of prominin-1 and inflammation arises from the observation that treatment of colon cancer in vivo or neuroblastoma cells in vitro with the nonsteroidal anti-inflammatory drug celecoxib or indomethacin decreased the amount of prominin-1–positive tumor cells [75]–[77]. Incidentally, an increased amount of enzyme cyclooxygenase-2 was observed in prominin-1–positive cells by comparison to negative ones [76], [77]. Therefore, the relation between these two molecules should be examined more closely through biochemical studies.

Interestingly, the co-expression analyses of cancerous tissues revealed that prominin-1 might complement, and eventually extend the clinical information gained with CEA and MUC1. For instance, all three markers showed similar expression patterns in PA, AciCC, AdCC and SA. The situation is significantly different in MEC, where only MUC1 shows a clear immunological signal. Previous studies have shown a relationship between MUC1 expression in MEC and the clinical outcomes [78]. Likewise, the MUC1 expression is an independent risk factor for predicting the recurrence of PA [79]. Indeed, Saores and colleagues have observed that MUC1 is more strongly expressed in carcinoma ex-PA by comparison to recurrent PA and PA [80]. Such observation suggests that MUC1 is valuable not only as a marker to predict recurrence, but also to monitor the malignant transformation. Similar investigation should be carried out with prominin-1.

We observed in the present histological samples that the secreted materials found in AdCC and PA were positive for CEA, MUC1 as well as prominin-1 suggesting that these membrane proteins are secreted. The partial association of CEA and MUC1 with saliva-derived prominin-1–containing membrane vesicles is coherent with such release. Given the presence of all three proteins (prominin-1, CEA and MUC1) in secreted materials mixed with the circulatory system, it is tempting to speculate that prominin-1–containing membrane vesicles may become a biomarker of AdCC and PA. Actually, an elevated level of CEA and MUC1 in blood can be used as an indicator of advanced forms of certain cancers [40]-[42], and the analysis of prominin-1–containing membrane vesicles in cerebrospinal fluids for diagnostic purposes is under investigation in view of their differential levels in relation with neural diseases [25], [26]. The physiological function of prominin-1–containing membrane vesicles is currently unknown, but the presence of micro-RNAs in melanoma-derived prominin-1–containing exosomes [81] suggests that they might play a role in intercellular communication and/or cancer progression as proposed earlier [28].

The release of prominin-1–containing membrane vesicles might occur by two independent pathways. They might derive from the tip of microvilli and primary cilium by budding and release as ectosomes as described in neural progenitors [21], [22] and intestinal cells [82]. The presence of microvilli at the apical domain of intercalated duct cells is consistent with such hypothesis. However, we should keep in mind that not all microvillar membranes contain prominin-1 as illustrated by its absence in striated ducts [19]. Such mechanism was also proposed for CEA-containing microvesicles [83]. Alternatively, prominin-1–containing membrane vesicles might come from the endocytic-exocytic pathway as exosomes, as recently described in hematopoietic stem cells [28]. The release of MUC1 in breast carcinoma cells and tracheobronchial epithelial cells seems to use this pathway [52], [84]. Yet, it cannot be excluded that both mechanisms occur simultaneously [28], [81]. The presence of prominin-1–negative membrane vesicles carrying ERM proteins indicates that multiple kinds of membrane vesicles are found in saliva. Indeed, a recent proteome of two distinct types of membrane vesicles that are classified according to their size and DPPIV (CD26) activity has shown that prominin-1 could be associated with both [23].

Within these extracellular membrane vesicles, we found that prominin-1 co-immunoprecipitates syntenin-1, a PDZ (PSD95/Dlg1/ZO-1) domain-containing scaffolding protein. Syntenin-1 interacts and controls the intracellular trafficking of a wide range of plasma membrane proteins [85] including those associated with tetraspanin-enriched microdomain [86], and is therefore directly or indirectly implicated in multiple physiological and pathological cellular processes. Together with syndecan and ALIX, syntenin-1 also regulates the biogenesis of exosomes [87], which is consistent with the release of prominin-1 in association with these vesicles. The ubiquitination of numerous proteins found in saliva-derived prominin-1–containing membrane vesicles and prominin-1 itself (Fig. 8B, C) are also in line with the presence of ubiquitinated proteins in exosomes as reported earlier for those found in human urine [88]. The nature, the location in relation with cellular trafficking and the significance of the interaction of prominin-1 with syntenin-1 need to be further determined, but the existence of various prominin-1 splice variants harboring distinct cytoplasmic C-terminal domains with alternative classes of PDZ-binding motif is interesting in the context of the modular and flexible recognition of PDZ domain-containing protein partner(s) [89]. Yet, in light of recent publications demonstrating the interaction of syntenin-1 with ubiquitin [90], [91], we could not exclude that syntenin-1 may somehow bind to the ubiquitin molecules linked to prominin-1. Such interaction might regulate the sorting of ubiquitinated prominin-1 into multi-vesicular bodies *en route* to exosomes. The isolation of a large prominin-1–containing protein complexes including syntenin-1 and CD63 needs also to be considered [24], [92], and the proteome of the immunoprecipitate will provide further insights. Given the growing body of publications suggesting a role for prominin-1 and syntenin-1 in cancer progression and metastasis their interaction under normal and pathological conditions deserves particular attention in a near future. It is worth mentioning in support of the present finding that both molecules were found in the proteomic profile of a sub-population of EpCAM-associated exosomes that are released from LIM1863 colon carcinoma cell-derived organoids [93].

In conclusion, the immunohistochemical profile of prominin-1 in human salivary glands reveals its general expression at the apical plasma membrane of the epithelial cells of intercalated ducts in normal glands, tumors and in inflammatory diseases such as SA. Biochemically, the ubiquitination of prominin-1 and its interaction with syntenin-1 highlight new features of its intra- and intercellular trafficking. Considering the link between stem cell differentiation and the loss of prominin-1 via asymmetric cell division and/or its release in association with membrane vesicles [16], [21], [28], [94], new perspectives in the biology of stem and cancer stem cells might emerge.

## Supporting Information

Figure S1 **Lack of prominin-1 in specific regions of pleomorphic adenoma, acinic cell and mucoepidermoid carcinomas.** PA (**A–C**), AciCC (**D**, **E**) and MEC (**F–K**) samples were labeled with 80B258 mAb directed against prominin-1 prior to hematoxylin counterstaining. Boxed areas (A1, B1, C1, D1, E1, E2) are displayed at higher magnification. In PA, mesenchymal-like components (i.e. chondroid, myxochondroid and hyalinized areas) are negative (A-C, respectively). In AciCC with solid growth pattern, black and white arrows indicate the lack prominin-1 in the acinar (D1, E1) and non-specific glandular (E2) cells, respectively. *CT*, connective tissue. In MEC, white, blue and black arrows indicate intermediate (F), squamous (G, H) and clear (I–K) cells, respectively. Histopathological characteristics of individual cases of PA: #5 (A), #3 (B) and #4 (C), AciCC: #2 (D) and #9 (E), and MEC: #14 (F), #6 (G, H), #9 (I–K) are summarized in Table S1. Scale bars 50 µm.
(TIF)

Figure S2 **prominin-1 is partially co-expressed with CEA and MUC1 in acinic cell carcinoma.** Consecutive sections of three individual cases of AciCC (**A–D**, see Table S1 for the histopathological characteristics) were immunolabeled for prominin-1 (80B258 mAb), CEA (Parlam 4) and MUC1 (115D8 or DF3) or with isotype control (ctrl) prior to hematoxylin counterstaining. Boxed areas (**B1**, **C1**) are displayed at higher magnification. Black arrowheads indicate partial (A, D) or complete (B1, C1) co-expression of analyzed antigens at the apical membrane of cells in the cyst-like structures present in the periphery of tumors (C, C1) or its individual nodules (A, B) and/or in the vicinity to hemorrhagic areas (C, C1, red asterisk). *CT*, connective tissue. Scale bars 50 µm.
(TIF)

Figure S3 **prominin-1 is co-expressed with either CEA or MUC1 or both in adenoid cystic carcinoma.** Consecutive sections of three individual cases of AdCC (**A–F**, see Table S1 for histopathological characteristics) were immunolabeled for prominin-1 (80B258 mAb), CEA (Parlam 4) and MUC1 (115D8 or DF3) or with isotype control (ctrl) prior to hematoxylin counterstaining. Boxed areas (**C1**) are displayed at higher magnification. Black arrowheads indicate the complete or partial co-expression of analyzed antigens in the duct-like structures that include the secretion (A) and the apical membrane of cells lining ducts (B, E, F). Blue arrowheads indicate immunoreactivities within the cytoplasm and/or entire plasma membrane of cells in solid tumor structures (B–D) present in the vicinity to the hemorrhagic areas (red asterisk). Red arrowheads show single tumor cells close to erythrocytes (C1). Note that the MUC1 detection by means of DF3 mAb is often weaker (C, D) or negative (E). Scale bars 50 µm.
(TIF)

Figure S4 **prominin-1 is partially co-expressed with CEA and MUC1 in pleomorphic adenoma, non-neoplastic peritumoral salivary gland regions and glands affected by sialadenitis.** Consecutive sections of PA (**A–C**), MEC (**D**) and SA (**E**, **F**) individual cases (see Table S1 for histopathological characteristics) were immunolabeled using 80B258, Parlam 4 and 115D8/DF3 mAbs directed against prominin-1, CEA and MUC1, respectively, or with isotype control (A**–**F, ctrl) prior to hematoxylin counterstaining. Two distinct areas of PA (A, B), peritumoral regions (C, D) and SA (E, F) are depicted. Dashed lines demarcate the tumor (*T*) from the surrounding non-neoplastic areas (blue asterisk). Black arrowheads indicate immunoreactivities at the apical membrane of cells lining either ductal structures of PA or intercalated ducts in both non-neoplastic peritumoral tissues (C, D) and SA (E, F). Note that DF3 antibody gives a weaker or negative signal in non-cancerous tissues. Scale bars 50 µm.
(TIF)

Figure S5 **MUC1, but not prominin-1 and CEA, is frequently expressed in mucoepidermoid carcinoma.** Consecutive sections of individual cases of MEC as indicated (**A–F**, see Table S1 for histopathological characteristics) were immunolabeled for prominin-1 (80B258 mAb), CEA (Parlam 4) and MUC1 (115D8 or DF3) or with isotype control (ctrl) prior to hematoxylin counterstaining. Orange arrowhead (A) points to prominin-1 in well-differentiated tumor mucous cells present in hemorrhagic region (red asterisk). MUC1 appears in squamous (C, D, blue arrow), intermediate (C, white arrow), clear (E, black arrow) and anaplastic (F, red arrow) tumor cells. MUC1 is found on the whole cell membrane or in cytoplasm (white and black arrowhead, respectively). Green arrowhead indicates CEA (F). Scale bars 50 µm.
(TIF)

Table S1 **Histopathological characteristics of individual cases.**
(DOC)

Table S2 **Specification of primary antibodies.**
(DOC)
