# Supplementary figures and images for: Human Prominin-1 (CD133) Is Detected in Both Neoplastic and Non-Neoplastic Salivary Gland Diseases and Released into Saliva in a Ubiquitinated Form
Source: PLoS One. 2014 Jun 9;9(6):e98927. doi: 10.1371/journal.pone.0098927 (PMC4050055; doi:10.1371/journal.pone.0098927)

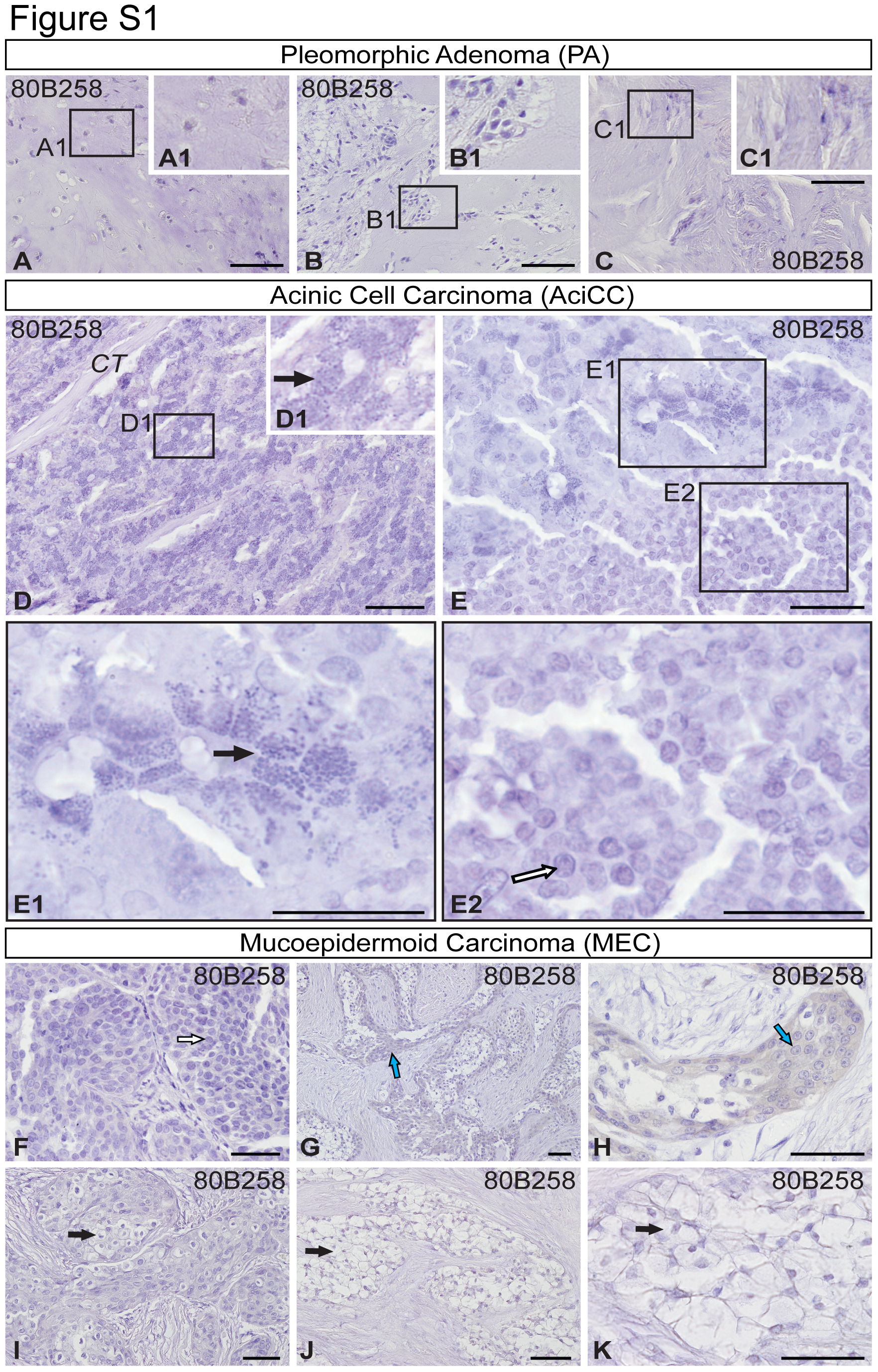

Supplement: Figure S1 — Lack of prominin-1 in specific regions of pleomorphic adenoma, acinic cell and mucoepidermoid carcinomas. PA (A–C), AciCC (D, E) and MEC (F–K) samples were labeled with 80B258 mAb directed against prominin-1 prior to hematoxylin counterstaining. Boxed areas (A1, B1, C1, D1, E1, E2) are displayed at higher magnification. In PA, mesenchymal-like components (i.e. chondroid, myxochondroid and hyalinized areas) are negative (A-C, respectively). In AciCC with solid growth pattern, black and white arrows indicate the lack prominin-1 in the acinar (D1, E1) and non-specific glandular (E2) cells, respectively. CT, connective tissue. In MEC, white, blue and black arrows indicate intermediate (F), squamous (G, H) and clear (I–K) cells, respectively. Histopathological characteristics of individual cases of PA: #5 (A), #3 (B) and #4 (C), AciCC: #2 (D) and #9 (E), and MEC: #14 (F), #6 (G, H), #9 (I–K) are summarized in Table S1. Scale bars 50 µm. (TIF) [file pone.0098927.s001.tif]

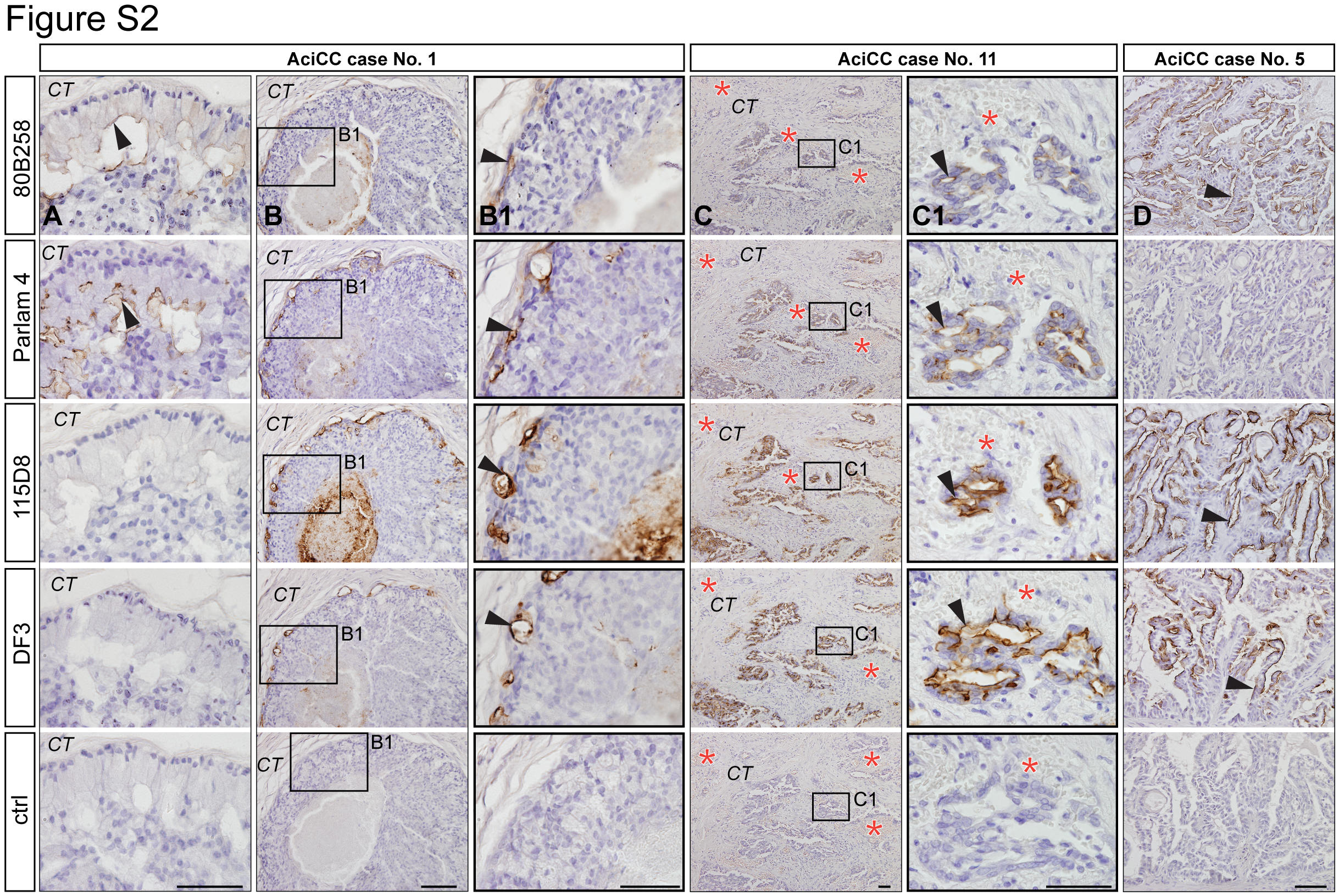

Supplement: Figure S2 — prominin-1 is partially co-expressed with CEA and MUC1 in acinic cell carcinoma. Consecutive sections of three individual cases of AciCC (A–D, see Table S1 for the histopathological characteristics) were immunolabeled for prominin-1 (80B258 mAb), CEA (Parlam 4) and MUC1 (115D8 or DF3) or with isotype control (ctrl) prior to hematoxylin counterstaining. Boxed areas (B1, C1) are displayed at higher magnification. Black arrowheads indicate partial (A, D) or complete (B1, C1) co-expression of analyzed antigens at the apical membrane of cells in the cyst-like structures present in the periphery of tumors (C, C1) or its individual nodules (A, B) and/or in the vicinity to hemorrhagic areas (C, C1, red asterisk). CT, connective tissue. Scale bars 50 µm. (TIF) [file pone.0098927.s002.tif]

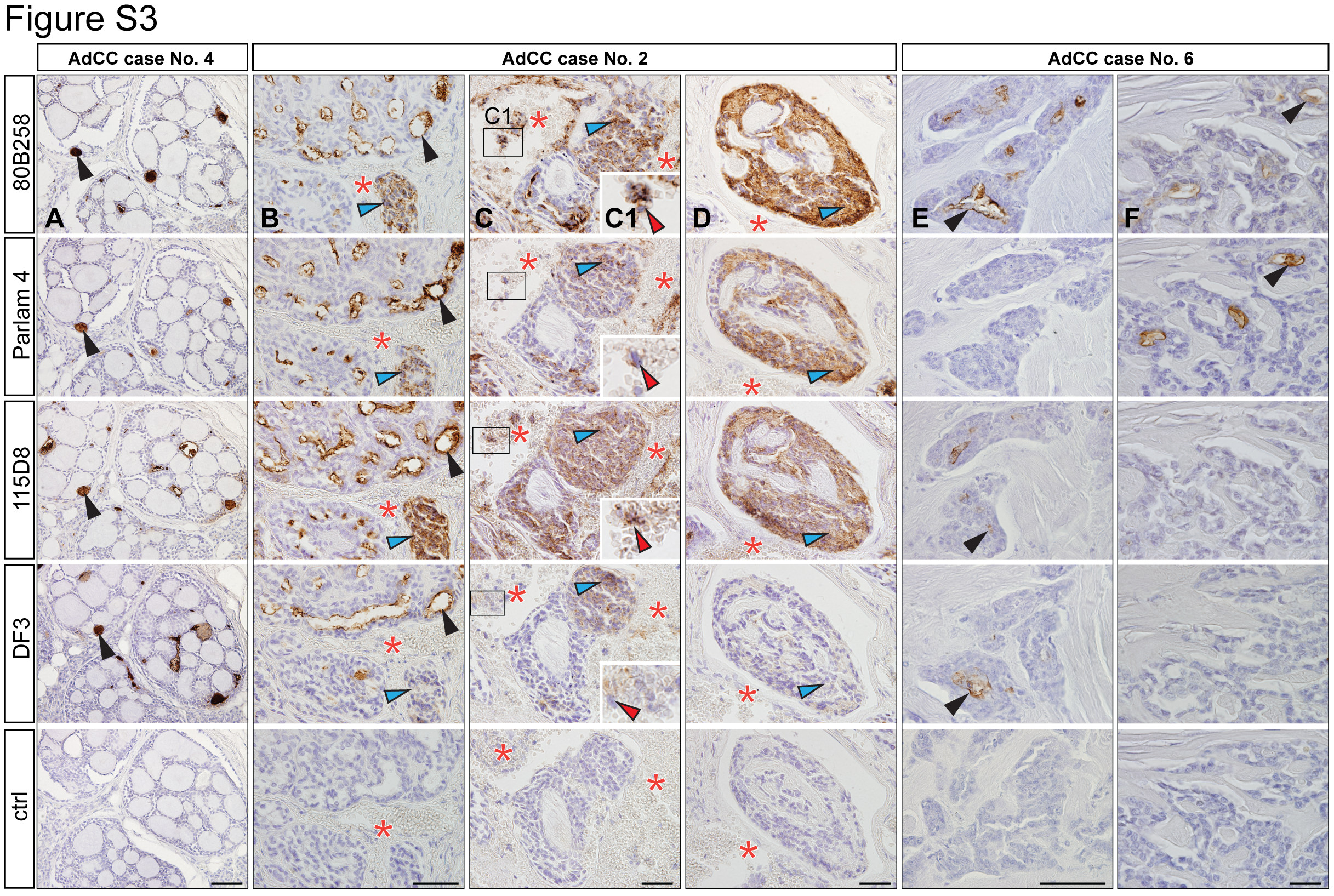

Supplement: Figure S3 — prominin-1 is co-expressed with either CEA or MUC1 or both in adenoid cystic carcinoma. Consecutive sections of three individual cases of AdCC (A–F, see Table S1 for histopathological characteristics) were immunolabeled for prominin-1 (80B258 mAb), CEA (Parlam 4) and MUC1 (115D8 or DF3) or with isotype control (ctrl) prior to hematoxylin counterstaining. Boxed areas (C1) are displayed at higher magnification. Black arrowheads indicate the complete or partial co-expression of analyzed antigens in the duct-like structures that include the secretion (A) and the apical membrane of cells lining ducts (B, E, F). Blue arrowheads indicate immunoreactivities within the cytoplasm and/or entire plasma membrane of cells in solid tumor structures (B–D) present in the vicinity to the hemorrhagic areas (red asterisk). Red arrowheads show single tumor cells close to erythrocytes (C1). Note that the MUC1 detection by means of DF3 mAb is often weaker (C, D) or negative (E). Scale bars 50 µm. (TIF) [file pone.0098927.s003.tif]

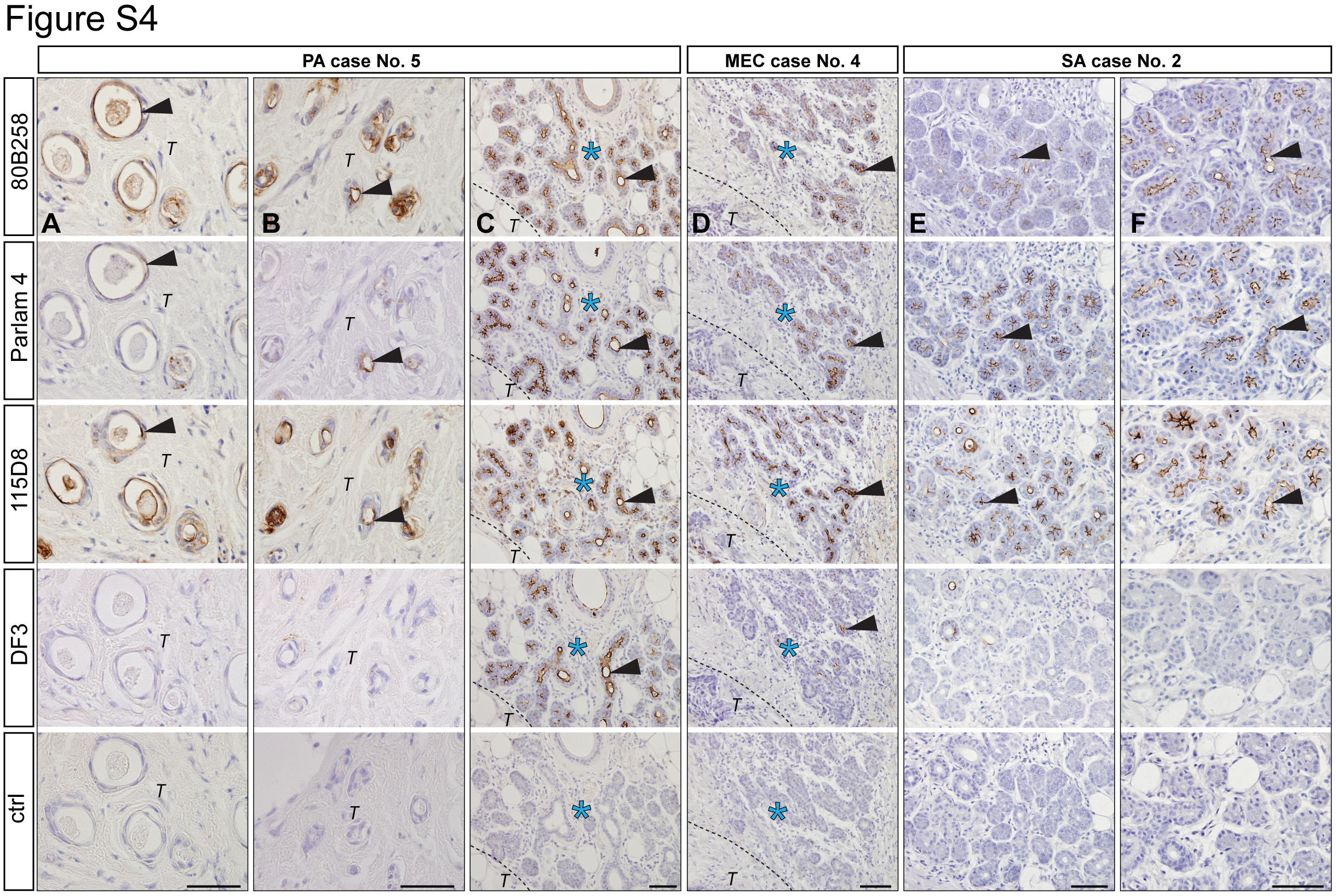

Supplement: Figure S4 — prominin-1 is partially co-expressed with CEA and MUC1 in pleomorphic adenoma, non-neoplastic peritumoral salivary gland regions and glands affected by sialadenitis. Consecutive sections of PA (A–C), MEC (D) and SA (E, F) individual cases (see Table S1 for histopathological characteristics) were immunolabeled using 80B258, Parlam 4 and 115D8/DF3 mAbs directed against prominin-1, CEA and MUC1, respectively, or with isotype control (A–F, ctrl) prior to hematoxylin counterstaining. Two distinct areas of PA (A, B), peritumoral regions (C, D) and SA (E, F) are depicted. Dashed lines demarcate the tumor (T) from the surrounding non-neoplastic areas (blue asterisk). Black arrowheads indicate immunoreactivities at the apical membrane of cells lining either ductal structures of PA or intercalated ducts in both non-neoplastic peritumoral tissues (C, D) and SA (E, F). Note that DF3 antibody gives a weaker or negative signal in non-cancerous tissues. Scale bars 50 µm. (TIF) [file pone.0098927.s004.tif]

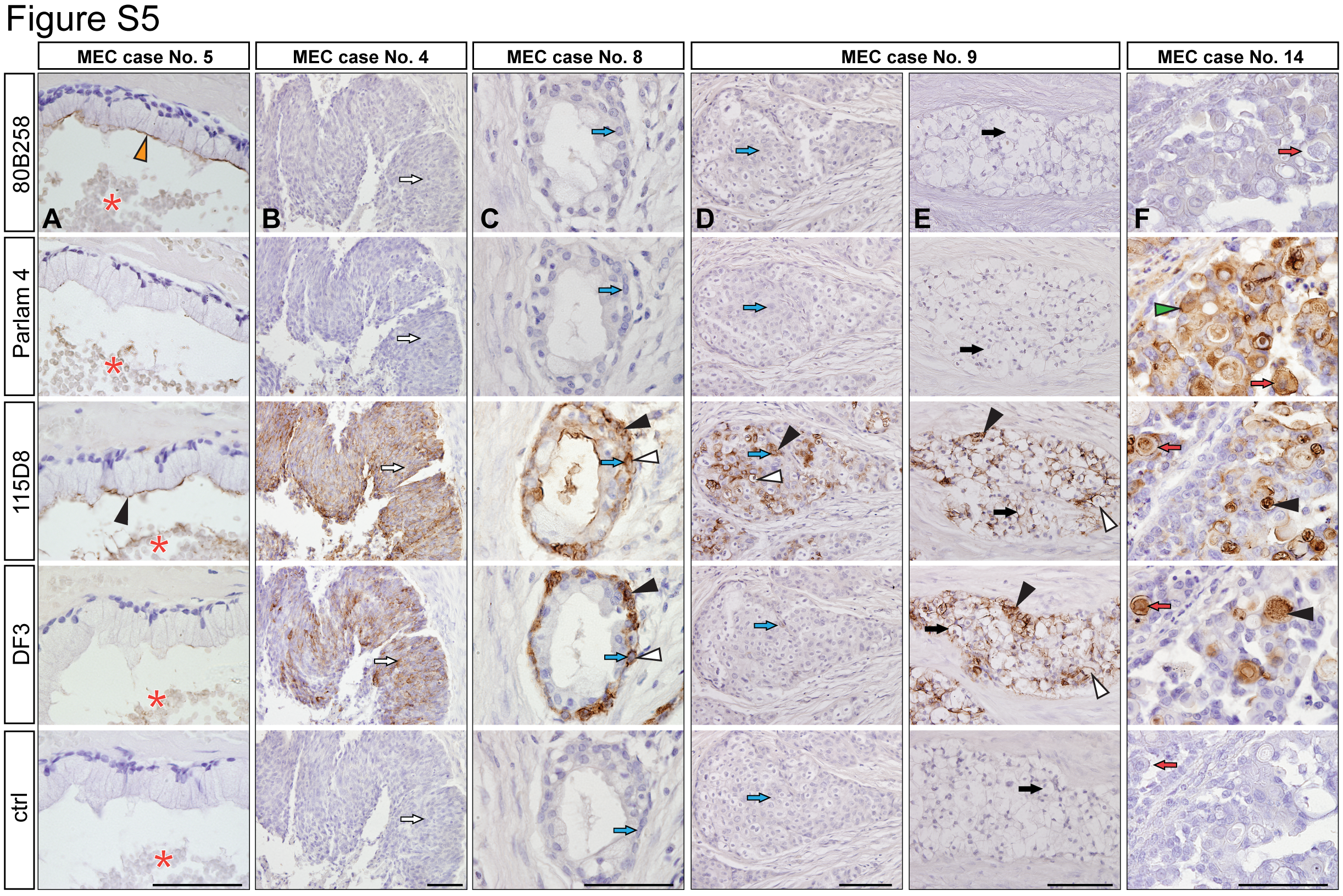

Supplement: Figure S5 — MUC1, but not prominin-1 and CEA, is frequently expressed in mucoepidermoid carcinoma. Consecutive sections of individual cases of MEC as indicated (A–F, see Table S1 for histopathological characteristics) were immunolabeled for prominin-1 (80B258 mAb), CEA (Parlam 4) and MUC1 (115D8 or DF3) or with isotype control (ctrl) prior to hematoxylin counterstaining. Orange arrowhead (A) points to prominin-1 in well-differentiated tumor mucous cells present in hemorrhagic region (red asterisk). MUC1 appears in squamous (C, D, blue arrow), intermediate (C, white arrow), clear (E, black arrow) and anaplastic (F, red arrow) tumor cells. MUC1 is found on the whole cell membrane or in cytoplasm (white and black arrowhead, respectively). Green arrowhead indicates CEA (F). Scale bars 50 µm. (TIF) [file pone.0098927.s005.tif]
