# Supplementary material for: Human Prominin-1 (CD133) Is Detected in Both Neoplastic and Non-Neoplastic Salivary Gland Diseases and Released into Saliva in a Ubiquitinated Form
Source: PLoS One. 2014 Jun 9;9(6):e98927. doi: 10.1371/journal.pone.0098927 (PMC4050055; doi:10.1371/journal.pone.0098927)
Supplement: Table S1 — Histopathological characteristics of individual cases. (DOC) [file pone.0098927.s006.doc]

**Table S1.** Histopathological characteristics of individual cases

| **Case** | **Sex^#^ /** **Age** | **Salivary gland^*^** | **TNM^§^** | **G** | **Re** | **Growth pattern^‡^** (predominant / additional) |
| --- | --- | --- | --- | --- | --- | --- |
| **Pleomorphic Adenoma (PA)** | | | | | | |
| 1 | M / 76 | P |  |  |  |  |
| 2 | F / 28 | P |  |  |  |  |
| 3 | M / 57 | P |  |  |  |  |
| 4 | F / 66 | P |  |  |  |  |
| 5 | M / 52 | P |  |  |  |  |
| 6 | M / 53 | P |  |  |  |  |
| 7 | F / 40 | P |  |  |  |  |
| 8 | F / 63 | P |  |  |  |  |
| 9 | F / 66 | P |  |  |  |  |
| 10 | M / 76 | P |  |  |  |  |
| **Acinic Cell Carcinoma (AciCC)** | | | | | | |
| 1 | M / 79 | P | T1 N0 M0 |  | 0 | S / MC |
| 2 | F / 78 | m/upper lip | T1 N0 M0 |  | 0 | S / MC |
| 3 | F / 71 | P | T1 N0 M0 |  | 0 | S / MC |
| 4 | F / 35 | P | T1 N0 M0 |  | 0 | PC |
| 5 | M / 55 | P | T1 N0 M0 |  | 0 | PC / F |
| 6 | M / 34 | P | T1 N0 M0 |  | 0 | S / MC |
| 7 | F / 70 | P | T1 N0 M0 |  | 1 | S / MC |
| 8 | F / 63 | SM | T2 N0 M0 |  | 0 | F / PC |
| 9 | F / 37 | P | T2 N0 M0 |  | 0 | S / MC |
| 10 | F / 34 | P | T2 N0 M0 |  | 0 | S / MC |
| 11 | M / 46 | P | T3 N0 M0 |  | 0 | MC |
| **Mucoepidermoid Carcinoma (MEC)** | | | | | | |
| 1 | M / 19 | m/hard palate | T1 N0 M0 | G1 | 0 |  |
| 2 | M / 55 | P | T1 N0 M0 | G1 | 0 |  |
| 3 | F / 47 | P | T1 N0 M0 | G1 | 0 |  |
| 4 | M / 69 | P | T1 N0 M0 | G2 | 0 |  |
| 5 | F / 40 | m/hard palate | T1 N0 M0 | G2 | 0 |  |
| 6 | M / 47 | P | T1 N0 M0 | G2 | 0 |  |
| 7 | M / 57 | m | T1 N0 M0 | G3 | 1 |  |
| 8 | M / 68 | P | T2 N0 M0 | G1 | 0 |  |
| 9 | M / 54 | SL | T2 N0 M0 | G3 | 0 |  |
| 10 | M / 62 | P | T2 N2b M0 | G3 | 1 |  |
| 11 | M / 81 | SM | T3 N0 M0 | G1 | 0 |  |
| 12 | F / 54 | P | T3 N0 M0 | G1 | 0 |  |
| 13 | M / 63 | P | T3 N2 M0 | G3 | 1 |  |
| 14 | F / 81 | m/floor of oral cavity | T3 N2a M0 | G3 | 1 |  |
| 15 | M / 70 | P | T3 N2b M0 | G3 | 1 |  |
| **Adenoid Cystic Carcinoma (AdCC)** | | | | | | |
| 1 | F / 28 | P | T1 N0 M0 |  | 0 | C / S |
| 2 | F / 74 | SM | T1 N0 M0 |  | 0 | C |
| 3 | F / 76 | m/upper lip | T1 N0 M0 |  | 0 | C |
| 4 | M / 32 | SM | T1 N0 M0 |  | 0 | C |
| 5 | M / 54 | SM | T2 N0 M0 |  | 0 | C |
| 6 | F / 55 | P | T2 N0 M0 |  | 1 | C |
| 7 | F / 58 | P | T4b N2b M1 |  | 1 | C |
| **Sialadenitis (SA)** | | | | | | |
| 1 | M / 34 | SM |  |  |  |  |
| 2 | M / 51 | SM |  |  |  |  |
| 3 | M / 53 | SM |  |  |  |  |

**^#^**M, male; F, female; age (year)

**^*^**m, minor; P, parotid; SL; sublingual; SM, submandibular

**^§^**TNM, classification of malignant tumors including size of the tumor (T), involving regional lymph nodes (N) and presence of distant metastases (M)

**^‡^**C, cribriform; F, follicular; MC, microcystic; PC, papillary-cystic; S, solid

G, histological grade; Re, recurrence
