# Supplementary material for: Human Prominin-1 (CD133) Is Detected in Both Neoplastic and Non-Neoplastic Salivary Gland Diseases and Released into Saliva in a Ubiquitinated Form
Source: PLoS One. 2014 Jun 9;9(6):e98927. doi: 10.1371/journal.pone.0098927 (PMC4050055; doi:10.1371/journal.pone.0098927)
Supplement: Table S2 — Specification of primary antibodies. (DOC) [file pone.0098927.s007.doc]

**Table S2.** Specification of primary antibodies

| **Antigen** | **Species^#^** | **Clone name or antiserum (as)** | **Source*** | **Dilution** | |
| --- | --- | --- | --- | --- | --- |
|  |  |  |  | IHC | WB |
| CEA | m | Parlam 4 | Acris | 1:25 | 1:200 |
|  | rb | as | Dako | 1:100 |  |
| CD63 | rb | as | Santa Cruz |  | 1:200 |
| Ezrin | rb | EP886Y | Abcam |  | 1:5000 |
| Flotillin-1 | m | 18 | BD Biosciences |  | 1:1000 |
| Flotillin-2 | m | 29 | BD Biosciences |  | 1:2000 |
| Ki67 | rb | as | Novocastra | 1:1000 |  |
| Moesin | rb | EP1863Y | Abcam |  | 1:5000 |
| MUC1 | m | 115D8 | Abcam | 1:50 | 1:200 |
|  | m | DF3 | GeneTex Inc. | 1:25 |  |
| Prominin-1 | m | AC133 | Miltenyi Biotec | 1:25 |  |
|  | rb | C24B9 | Cell Signaling |  | 1:1000 |
|  | m | 80B258 | Ref. [19]^§^ | 1:200 | 1:2000 |
| Radixin | rb | EP1862Y | Abcam |  | 1:5000 |
| Syntenin-1 | rb | as | Abcam |  | 1:5000 |
| Ubiquitin | rb | as | Dako |  | 1:1000 |

IHC, immunohistochemistry; WB, immunoblotting

**^#^** m, mouse; rb, rabbit

*Abcam (Cambridge, UK), Acris Antibodies GmbH (Herford, Germany), BD Biosciences (Heidelberg, Germany), Cell Signaling Technology (Danvers, MA), Dako (Glostrup, Denmark), GeneTex Inc. (Irvine, CA), Miltenyi Biotec (Bergisch Gladbach, Germany), Novocastra Laboratories (Newcastle, UK), Santa Cruz Biotechnology, Inc. (Santa Cruz, CA), Sigma (Dartmouth, Germany)

^§^See main text
